# Polysaccharides from Er Chen Decoction: gastrointestinal fate, gut microbiota modulation and metabolomics

**DOI:** 10.3389/fmicb.2026.1931914

**Published:** 2026-09-15

**Authors:** Yu-Jie Liu, Zhi-Kun Wang, Lian-Tao Yue, Dan Qiao, Qiao-Ling Deng, Xiao-Ying Chang, Tong Liu, Mei-Bian Hu

**Affiliations:** School of Traditional Chinese Medicine and Food Engineering, Shanxi Provincial Key Laboratory of Traditional Chinese Medicine Processing, Shanxi University of Chinese Medicine, Jinzhong, China

**Keywords:** Er Chen Decoction, fermentation, gut microbiota, metabolomics, polysaccharides

## Abstract

**Background:**

Er Chen Decoction (ECD), a classical formula for phlegm-related disorders, contains polysaccharides (PECD) as an important bioactive fraction. This study systematically assessed their gastrointestinal absorption, digestive stability, fecal fermentation, and correlations with gut microbiota and metabolic changes.

**Methods:**

*In vivo* gastrointestinal absorption of PECD were evaluated using fluorescence tracing of fluorescein isothiocyanate (FITC)-labeled polysaccharides. The stability of PECD during simulated oral, gastric, and intestinal digestion was assessed by determining total/reducing sugar contents, molecular weight distribution, monosaccharide composition, and Fourier transform infrared spectra. Subsequently, *in vitro* fecal fermentation of PECD was evaluated by pH, short-chain fatty acids (SCFAs), 16S rRNA gene sequencing, and untargeted metabolomics.

**Results:**

No appreciable systemic absorption of FITC-labeled PECD was detected following gastrointestinal administration. During simulated digestion, the total sugar and reducing sugar contents, molecular weight distribution, monosaccharide composition, and infrared spectral profiles of PECD showed no significant changes, suggesting that its major polysaccharides were largely resistant to upper gastrointestinal digestion. During *in vitro* fecal fermentation, PECD was progressively utilized by the gut microbiota, accompanied by a reduction in pH and an increase in SCFAs concentrations. PECD supplementation was also associated with shifts in microbial community composition by enriching genera such as *Bifidobacterium* and *Mitsuokella*, while depleting others including *Klebsiella* and *Bilophila*. Untargeted metabolomic analysis revealed alterations in metabolites related to phenylalanine metabolism, caffeine metabolism, and arginine biosynthesis, suggesting their potential involvement in microbial response to PECD.

**Conclusion:**

PECD exhibited limited absorption, resistance to upper gastrointestinal digestion, susceptibility to fecal microbial fermentation, which altered microbial composition, SCFAs, and metabolic profiles, supporting its further study as a candidate bioactive polysaccharide.

## 1 Introduction

Er Chen Decoction (ECD) is a classic formula in Traditional Chinese Medicine (TCM) for treating phlegm syndrome. Its origin can be traced back to the *Prescriptions of the Bureau of Taiping People's Welfare Pharmacy*, a classic medical text compiled during the Song Dynasty. ECD consists of six medicinal herbs, as follows: *Pinellia Rhizome* (Banxia), *Pericarpium Citri Reticulatae* (Chenpi), *Poria* (Fuling), *Glycyrrhizae Radix Et Rhizoma Praeparata Cum Melle* (Zhigancao), *Mume Fructus* (Wumei), and *Zingiberis Rhizoma Recens* (Shengjiang) (Wang et al., 2025). ECD is traditionally prescribed to dispel dampness, eliminate phlegm, regulate qi movement, and restore middle-jiao harmony. Its principal herbal components, including Zhibanxia, Chenpi, Fuling, and Zhigancao, work in concert to produce these therapeutic actions, combined with Shengjiang and Wumei to reduce toxicity and correct deviation. In clinical practice, doctors have studied and observed the disease efficiency of 84 patients with bronchial asthma divided into two groups. The study results demonstrated that the overall efficacy rate of ECD reached 95.3%, which can effectively improve the symptoms and signs of the body and restore immune regulation (Bao et al., 2019). A total of 438 individuals were randomly assigned to either the experimental condition or the control condition. Baseline assessments indicated that the two groups were comparable in total cholesterol, triglyceride levels, low-density lipoprotein cholesterol, body mass index, and visceral fat area, with no statistically significant differences observed (all *p* > 0.05). Following treatment, compared with the control group, the experimental group exhibited significantly superior improvement across all evaluated parameters. These results indicate that ECD, as an adjuvant therapy for obesity, may facilitate clinical recovery and promote the attenuation of multiple metabolic risk factors (Lv and Kang, 2024).

Research has shown that many polysaccharides from TCM can safely and effectively treat lung diseases and metabolic disorders (Jiang W. et al., 2024; Liu Y. J. et al., 2024). However, most of the attention has been paid to polysaccharides from single medicinal materials, with limited attention given to those derived from compound formulas. Available evidence indicates that, relative to individual bioactive polysaccharides, compound polysaccharides exhibit superior biological activity in both magnitude and breadth, particularly with respect to immunomodulatory effects (Xu and Zhang, 2025). For instance, a polysaccharide formulation containing *Agaricus blazei* Murrill, *Grifola frondosa*, and *Pericarpium Citri Reticulatae* demonstrated enhanced antitumor activity and markedly decreased the viability of MC38-N4 cells (Xu et al., 2024). Another study also reported the combined immunomodulatory effects of lentinan, pachymaran, and *Tremella* polysaccharides were more pronounced than those produced by any single polysaccharide alone (Luo et al., 2018). Moreover, Deng et al. systematically characterized the synergistic properties of a seven-component herbal polysaccharide system derived from *Lentinula edodes, Ganoderma lucidum, Tremella fuciformis*, chrysanthemum, *Lycium barbarum, Codonopsis pilosula*, and *Poria cocos* at an equal ratio (1:1:1:1:1:1:1). Their findings provide a useful foundation for evaluating the quality and optimizing the formulation of compound polysaccharides in biomedical and functional food applications (Deng et al., 2020). Unlike single-herb polysaccharides, formula-derived polysaccharides are obtained through the co-decoction and co-extraction of multiple constituent herbs, a process that may induce intermolecular interactions, structural rearrangements, and the formation of novel complexes that are absent in single-herb extracts (Huang et al., 2022; Nie et al., 2025). Consequently, the digestive behavior, microbial fermentability, and biological activity of formula-derived polysaccharides cannot be directly extrapolated from those of single-herb counterparts. While several single-herb polysaccharides from individual components of the ECD formula have been reported (Wu et al., 2022; He and Yang, 2023; Ye et al., 2023; Li X. et al., 2024), no systematic study has addressed the polysaccharide fraction derived from the whole formula. Moreover, investigating the whole-formula polysaccharides is clinically more relevant, as it reflects actual TCM prescribing practices in which multiple herbs are used in combination. To address this gap, the present study focused on PECD, a polysaccharide fraction extracted from the complete ECD formula, and systematically investigated its physicochemical properties, gastrointestinal stability, and gut microbiota-modulating potential via *in vitro* fermentation and metabolomics.

Growing evidence suggests that the biological activity of polysaccharides appears to be strongly associated with their intrinsic physicochemical properties, especially molecular weight, monosaccharide constituents, and glycosidic bond configurations. Critically, the gastrointestinal milieu characterized by variable acidity, bile salts, and digestive enzymes, which can structurally alter these polysaccharide attributes during digestion (Gao et al., 2024). Meanwhile, upon reaching the large intestine, polysaccharides undergo microbial fermentation, resulting in the production of advantageous metabolites like short-chain fatty acids (SCFAs). This production of SCFAs contributes to enhanced intestinal homeostasis. Consequently, systematic evaluation of structural and compositional alterations in polysaccharides throughout gastrointestinal digestion and colonic fermentation is essential for elucidating how they function *in vivo*, determining their potential biofunctional properties, and characterizing their bioavailability (Fang et al., 2022). Increasing evidence has demonstrated that gut microbiota dysregulation is strongly correlated with the pathogenesis of various diseases, including inflammatory, metabolic, and immune-related disorders. Such microbial disturbances may initiate or exacerbate disease progression through multiple pathways. SCFAs are recognized as the major metabolites generated by the gut microbiota, crucial for maintaining the intestinal epithelial cell barrier, regulating host immune responses, and maintaining oxidative antioxidant balance. Modulation of gut microbiota homeostasis and its metabolites, particularly SCFAs, may represent a promising therapeutic strategy for a wide range of diseases (Ma et al., 2021). In addition, polysaccharides help maintain intestinal homeostasis through multiple actions, including improvement of gut health, promotion of beneficial microbial growth, inhibition of pathogenic bacteria, and regulation of gut microbial community composition (Li J. et al., 2024). Therefore, sequential investigation of the structural changes, microbial utilization, and metabolite production of polysaccharides during simulated gastrointestinal digestion and colonic fermentation is essential for elucidating their potential gut microbiota-modulatory effects (Zhang et al., 2022; Xue et al., 2024). At present, the physicochemical characteristics, digestive stability, and microbial fermentation behavior of PECD remain insufficiently characterized. Our previous study investigated the *in vivo* distribution, simulated gastrointestinal digestion, fecal fermentation properties, and effects on the human gut microbiota of polysaccharides isolated from alum-processed *Pinellia* rhizome (Gao et al., 2024). Unlike polysaccharides derived from a single medicinal material, PECD is obtained through the co-decoction and co-extraction of multiple constituent herbs. Its polysaccharide components therefore originate from more diverse sources and may exhibit greater compositional heterogeneity and more complex digestive and fermentative behavior (Jang et al., 2025; Xu and Zhang, 2025).

Accordingly, the present study systematically investigated the gastrointestinal distribution and digestive stability of PECD, its microbial fermentability, and the associated changes in microbial communities and metabolic profiles. We further examined the associations among differential microbial taxa, SCFAs, and other differential metabolites. This integrated approach provides a basis for understanding the formula-derived characteristics of PECD, generates experimental evidence for evaluating its candidate prebiotic properties, and supports further investigation of the previously underappreciated polysaccharide components in TCM decoctions and their potential roles in gut microbiota regulation.

## 2 Materials and methods

### 2.1 Materials and chemicals

The composition of ECD is presented in Table 1. Qingbanxia was sourced from Sichuan Neautus Traditional Chinese Medicine Co., Ltd. (Sichuan, China). Chenpi Fuling, and Zhigancao were supplied by Shanxi Weikangtang Traditional Chinese Medicine Slices Co., Ltd. (Shanxi, China). Wumei was purchased from Dashenlin Pharmaceutical Group Co., Ltd., whereas Shengjiang was sourced from a local market in Jinzhong, China. Fluorescein isothiocyanate (FITC) was acquired from ChemeGen Biotechnology Co., Ltd. (Shanghai, China). Monosaccharide reference standards, specifically mannose (Man), rhamnose (Rha), galactose (Gal), galacturonic acid (GalA), glucose (Glc), glucuronic acid (GlcA), fucose (Fuc), xylose (Xyl), and arabinose (Ara), were products of the National Institute for Food and Drug Control (Beijing, China). Pepsin, trypsin, and pancreatic enzymes came from Sigma-Aldrich Co., Ltd. (St. Louis, MO, USA). Peptone and yeast extract were procured from Beijing Solarbio Science & Technology Co., Ltd. (Beijing, China). SCFA standards, including acetic acid, propionic acid, isobutyric acid, n-butyric acid, isovaleric acid, and n-valeric acid, were bought from McLean Biochemical Technology Co., Ltd. (Shanghai, China). α-Amylase and MD44 dialysis bags (3,000 Da) were supplied by Yuanye Bio-Technology Co., Ltd. (Shanghai, China). All other chemicals of analytical grade were sourced from Kermel Chemical Reagents Co., Ltd. (Tianjin, China).

**Table 1:** Er Chen Decoction composition.

Chinese name	Latin name	Botanical plant name	Part used	Weight (g)
Qingbanxia	*Pinelliae Rhizoma Praeparatum Cum Alumine*	*Pinellia ternata* (Thunb.) Breit.	Tuber	15
Chenpi	*Pericarpium Citri Reticulatae*	*Citrus reticulata* Blanco	Fruit peel	15
Fuling	*Poria*	*Poria cocos* (Schw.) Wolf	Sclerotium	9
Zhigacao	*Glycyrrhizae Radix Et Rhizoma Praeparata Cum Melle*	*Glycyrrhiza uralensis* Fisch.	Rhizomes	4.5
Wumei	*Mume Fructus*	*Prunus mume* (Sieb.) Sieb. et Zucc.	Fruit	9
Shengjiang	*Zingiberis Rhizoma Recens*	*Zingiber officinale* Rosc.	Rhizome	3

### 2.2 Preparation of PECD

Polysaccharides derived from ECD were extracted following the procedure described below. Powdered herbal material (as shown in Table 1) was defatted with 95% ethanol (1:8, w/v) under reflux for 1 h. The obtained residue was dried and then extracted twice by reflux with distilled water (1:10) for 1 h each time under reflux. After filtration of the aqueous extract, the collected filtrate was concentrated under vacuum. To precipitate the polysaccharides, the sample was treated with 95% ethanol at a 1:4 (v/v) ratio, and the mixture was subsequently left to stand at 4 °C overnight. The obtained precipitate was collected, washed extensively with absolute ethanol, and dried at 50 °C to yield the crude PECD fraction. The dried polysaccharide material was re-dissolved, and α-amylase was added to the solution (20 U/mL). The enzymatic reaction was conducted at 40 °C for 16 h under constant agitation to ensure complete starch hydrolysis. The completeness of starch degradation was verified using an iodine-potassium iodide (I_2_-KI) test solution. The sample was boiled to stop the enzymatic reaction, and followed by centrifugation. The collected supernatant was dialyzed against distilled water using a membrane with a nominal dialysis bag of 3 kDa for 48 h, and the resulting purified polysaccharide solution was freeze-dried for long-term preservation. The extraction yield of PECD was 2.12 ± 0.16% (w/w) based on the dried starting material. The total carbohydrate content determined by the phenol-sulfuric acid method was 68.45 ± 0.5%, representing the purity of the polysaccharide fraction. The protein content was 0.68 ± 0.15% (BCA assay), and UV scanning showed only weak absorbance in the 260–280 nm region, suggesting negligible protein/nucleotide contamination in the final product. Structural characterization analysis on the prepared PECD samples according was performed using the methods we previously reported (Matou et al., 2023; Gao et al., 2024). The structural characteristics of PECD were systematically characterized by determining its molecular weight, monosaccharide composition, Fourier transform infrared (FT-IR) spectra, and Polysaccharide Analysis using Carbohydrate gel Electrophoresis (PACE). The results were shown in Supplementary Figure S1.

### 2.3 *In vivo* distribution of FITC-labeled PECD in mice

#### 2.3.1 FITC-labeled PECD (FPECD) preparation

The FITC conjugation of PECD was carried out conducted using the procedure established by Lin et al. in a prior study (Lin et al., 2011). Briefly, using dimethyl sulfoxide spiked with a trace of pyridine, a PECD solution was formulated to a concentration of 100 mg/mL. Fluorescein isothiocyanate (100 mg) and dibutyltin dilaurate (20 μL) were then added, and mixed thoroughly. The reaction mixture maintained at 95 °C for a period of 2 h. Upon completion, precipitation of the product was achieved by adding absolute ethanol at a 1:4 (v/v) volume ratio, and the precipitation procedure was repeated four times to remove residual unbound dye. Further purification of the resulting product was achieved by dialysis using a 3,000 Da molecular weight cut-off membrane. Absence of visible fluorescence in the final dialysate under UV illumination was used as an operational check for removal of unbound FITC.

FITC conjugation to PECD was quantified spectrophotometrically at 490 nm using a FITC calibration curve. Briefly, a FITC stock solution was prepared in dimethyl sulfoxide and serially diluted with phosphate-buffered saline (PBS, pH 7.4) to obtain a series of standard solutions spanning the expected concentration range of the samples. All standards and samples were prepared in the same solvent system and adjusted to the same final pH. Unlabeled PECD at matching concentration served as blank to correct for background absorbance and scattering. All measurements were performed in triplicate under light-protected conditions. The FITC labeling efficiency of PECD was calculated as 1.01 ± 0.01% via the following Equation 1.


$$\begin{array}{c} \text{FITC labeling efficiency of PECD }\left(\text{\%}\right)\;=\;\frac{{\text{C}}_{\text{FITC}}\times \text{V}}{{\text{m}}_{\text{FITC},\text{ added}}}\;\times \;100\% \end{array}$$ (1)

where C_FITC_ is the FITC-equivalent concentration in the purified FITC–PECD solution (μg/mL), V is the final volume of the purified FITC–PECD solution (mL), and m_FITC, added_ is the initial mass of FITC added to the labeling reaction (mg).

#### 2.3.2 Dynamic *in vivo* distribution of FPECD in mice

All procedures involving animals were carried out in compliance with established experimental regulations and received approval from the Experimental Animal Ethics Committee of Shanxi University of Chinese Medicine. Controlled environmental conditions were provided for Kunming (KM) mice (20 ± 2 g), including an ambient temperature of 22 ± 2 °C, relative humidity of 55 ± 10%, and a 12-h alternating light/dark cycle. The animals were divided randomly into seven groups, each consisting of three individuals (*n* = 3), including one blank control group and six treatment groups corresponding to different time points (0, 1, 2, 3, 4, and 6 h). Mice assigned to the treatment groups were administered 4 mg of FPECD by oral gavage at a final volume of 0.3 mL, whereas animals assigned to the blank control group received an equivalent volume of normal saline. At each designated time point, painless and humane euthanasia was performed on animals by intraperitoneal injection of a lethal dose of sodium pentobarbital (150 mg/kg). Surgery was performed to open the chest and abdomen of mice, and whole-body fluorescence images were obtained with a GelView 6000 Pro II multifunctional imaging system (Guangzhou Biolight Biotechnology Co., Ltd., Guangzhou, China). Imaging was performed with excitation at 475 nm, emission at 535 nm, exposure time of 5 s, light-emitting diode (LED) intensity at 100%, pixel binning of 4 × 4, aperture *f* /0.95, and charge-coupled device (CCD) gain at software default. These identical acquisition settings were applied across all animals and imaging time points. Subsequently, the stomach, lungs, small intestine, cecum, colon, liver, spleen, and kidneys were removed to perform *ex vivo* fluorescence imaging. Fluorescence intensities were expressed as relative fluorescence units (RFU) measured from regions of interest (ROIs) of identical size and anatomical location without additional normalization, because all acquisitions were performed under uniform conditions.

### 2.4 Simulated gastrointestinal digestion of PECD *in vitro*

An *in vitro* gastrointestinal digestion procedure was carried out based on the method reported previously (Gao et al., 2024). This digestive model consisted of three sequential steps (oral, gastric, and small intestinal), which together replicated the *in vivo* digestive process (Matou et al., 2023).

#### 2.4.1 *In vitro* salivary digestion

The *in vitro* salivary digestion procedure for PECD was conducted based on a previously published method (Gao et al., 2024). Briefly, to prepare the artificial saliva, 430 mg α-amylase, 760 mg NaCl, 1.49 g KCl, and 130 mg CaCl_2_·2H_2_O were added to 1 L of distilled water, after which the pH was adjusted to 6.8. Subsequently, Equal volumes of PECD solution (300 mL, 4 mg/mL) and simulated saliva were mixed and incubated at 37 °C in a thermostatically controlled shaker. Throughout digestion, 10 mL samples were collected at 0, 5, 15, and 30 min, and each aliquot was immediately heated in boiling water for 10 min to stop enzymatic activity. Simultaneously, 50 mL portions collected at the same intervals were precipitated with absolute ethanol (1:4, v/v). The precipitated materials were then re-dissolved, dialyzed using a molecular weight cut-off of 3,000 Da membrane, and freeze-dried. These products, generated after the salivary digestion process, were designated PECD-S.

#### 2.4.2 *In vitro* gastric digestion

To prepare the simulated gastric electrolyte solution, a mixture containing 3.1 g NaCl, 0.6 g NaHCO_3_, 1.1 g KCl, and 0.15 g CaCl_2_·2H_2_O was dissolved in 1 L of deionized water. Following dissolution, HCl was added to adjust the pH to 3.0. Subsequently, the simulated gastric electrolyte solution (300 g) received the addition of 354 mg of gastric protease together with 3.0 mL of 1 M sodium acetate solution (pH 5.0). The mixture was subjected to magnetic stirring for 15 min to obtain a homogeneous system, followed by readjustment of the pH to 3.0. The gastric digestion phase was initiated by introducing 360 mL of the prepared simulated gastric juice after 30 min of salivary digestion. For the blank control, deionized water substituted the salivary digestion reaction mixture. Sampling (10 mL each) was performed at 0, 0.5, 1, 2, 4, and 6 h, with immediate termination of enzymatic activity by boiling for 10 min. In parallel, 50 mL specimens were taken at the same time points, subjected to the same processing as the samples collected after salivary digestion, and designated PECD-G (salivary-gastric digestion samples).

#### 2.4.3 *In vitro* small intestinal digestion

To prepare the intestinal electrolyte solution, 5.4 g NaCl, 0.65 g KCl, and 0.33 g CaCl_2_·2H_2_O, were dissolved in 1 L of deionized water, after which 0.1 M NaOH was added to bring the pH to 7.0. Bile salt and pancreatic enzyme solutions were individually formulated in deionized water at concentrations of 4% and 7% (w/v), respectively. The simulated intestinal fluid was formulated by mixing 100 g of intestinal electrolyte solution with bile salt solution (200 g), pancreatic enzyme solution (100 g), and trypsin (13 mg) until a homogeneous mixture was obtained. Thereafter, the pH of the prepared mixture was brought to 7.5 with 0.1 M NaOH. Following the simulated gastric digestion step, the resulting digesta was neutralized to pH 7.0 by the addition of 1 M NaHCO_3_. The simulated small-intestinal digestion stage was then started by introducing 108 mL of the pre-prepared simulated intestinal fluid. During the incubation period, 10 mL aliquots were withdrawn at preselected time intervals (0, 0.5, 1, 2, 4, and 6 h) for subsequent analysis. Following collection, the samples were promptly exposed to boiling water for 10 min to achieve enzyme inactivation. At the corresponding sampling time points, additional 50 mL aliquots were simultaneously obtained. These larger aliquots were processed identically to samples after the salivary digestion (as described in the corresponding method section) and designated as PECD-I (samples obtained after salivary, gastric, and intestinal digestion).

#### 2.4.4 PECD sample preparation following simulated digestion

Upon completion of the simulated intestinal digestion, the remaining digestive fluid was subjected to boiling water for 10 min to inactivate the digestive enzymes. The resulting mixture was then centrifuged, followed by dialysis (3,000 Da cut-off) for 48 h. Finally, the dialyzed product was freeze-dried to yield the digested form of PECD.

#### 2.4.5 Carbohydrate content determination

Total sugar and reducing sugar levels during different digestion stage were quantified using the phenol-sulfuric acid and 3,5-dinitrosalicylic acid methods, respectively, with D-glucose as the standard.

#### 2.4.6 Molecular weight determination

An UltiMate U-3000 HPLC system (Thermo Fisher Scientific, USA) coupled with an evaporative light-scattering detector was employed for the analysis of the different samples. Separation took place on a TSK Gel G4000 SWXL column (7.8 mm × 300 mm; Tosoh Corporation, Japan), with ultrapure water as the mobile phase flowing at 0.7 mL/min. The column was held at 30 °C throughout the analysis, and the injection volume for each sample was set to 10 μL.

#### 2.4.7 Monosaccharide composition analysis

The prepared samples were subjected to derivatization with 1-phenyl-3-methyl-5-pyrazolone (PMP) under an alkaline medium. The PMP-labeled monosaccharide derivatives were then separated on a ultra-high-performance liquid chromatography (UHPLC) system (UltiMate 3000, Thermo Fisher Scientific, Waltham, MA, USA) fitted with a UV detector. A hydrophilic ODS-5 C_18_ column (4.6 mm inner diameter × 250 mm length, 5 μm particle size; Beijing H&E Co., Ltd., Beijing, China) thermostated at 30 °C was used for separation. The mobile phase consisted of acetonitrile and 0.05 mol/L phosphate-buffered saline (pH 6.8) mixed at an 18:82 ratio (v/v), delivered isocratically at 1.0 mL/min. The injection volume was 10 μL per run, and UV absorbance was monitored at 245 nm.

#### 2.4.8 FT-IR analysis

Samples were homogenized with spectroscopic-grade potassium bromide (KBr) powder at a ratio of approximately 1:100 (sample:KBr, w/w) and compressed into transparent pellets (1 mm thickness) under vacuum. Infrared spectral data were acquired using a Nicolet iS10 Fourier transform infrared spectrometer (Thermo Fisher Scientific, Waltham, MA, USA) fitted with a DTGS detector. Spectral data were acquired in the 4,000–400 cm^−1^ region with a resolution setting of 4 cm^−1^. For each sample, 32 scans were averaged to obtain the final spectrum. Background spectra of pure KBr pellets were recorded and subtracted prior to sample.

### 2.5 Simulated *in vitro* fermentation of PECD

*In vitro* simulated gastrointestinal fermentation was performed using a protocol reported in a previous study (Tian et al., 2021; Gao et al., 2024). The procedure involved preparation of the fermentation medium, generation of the microbial suspension, and *in vitro* simulated fermentation to mimic the fermentation process.

#### 2.5.1 Preparation of the fermentation medium

The basic cultivation medium was prepared with deionized water, and the total volume was adjusted to 1.0 L. A basal medium was formulated with the following composition: peptone (2.0 g/L), yeast extract (2.0 g/L), bile salts (0.5 g/L), L-cysteine hydrochloride (0.5 g/L), sodium chloride (100 mg/L), sodium bicarbonate (2.0 g/L), KH_2_PO_4_ (40 mg/L), K_2_HPO_4_ (40 mg/L), MgSO_4_·7H_2_O (10 mg/L), CaCl_2_·6H_2_O (10 mg/L), and heme chloride (20 mg/L). In addition, Tween 80 and vitamin K1 were included at 2.0 mL/L and 10 μL/L, respectively, whereas resazurin solution (1.0 mL/L, 1.0%, w/v) was incorporated as an indicator for anaerobic conditions. The pH of the prepared medium was first adjusted to 7.0 with 0.1 M HCl. It was then sterilized by autoclaving at 121 °C for 15 min. To obtain the treatment media, PECD and FOS were first dispensed separately into sterile culture dishes and subsequently mixed with the sterilized basal medium, yielding the PECD treatment medium and the FOS positive-control medium, respectively.

#### 2.5.2 Generation of the microbial suspension

All fermentation assays were conducted in three independent biological replicates. Fresh fecal specimens were collected from six healthy adults (20–30 years old), of whom three were male and three were female. The participants were characterized by habitual dietary patterns, an absence of gastrointestinal disorders, and no antibiotic use in the 3 months before sample collection. Equal wet weights of fresh feces from each donor were homogenized and pooled to generate the mixed inoculum. This pooled design was chosen to reduce inter-individual variability and provide a more representative and standardized microbial community for initial screening of PECD's fermentability. The mixture was fully homogenized and immediately resuspended in a sterile modified saline medium composed of 0.5 g/L L-cysteine hydrochloride and 9.0 g/L NaCl, thereby producing a 20% (w/v) fecal slurry. After being mixed well, the suspension was filtered through a sterile gauze layer, and the collected filtrate was maintained inside an anaerobic chamber before being used further. This study was approved by the Medical Ethics Committee of Shanxi University of Chinese Medicine.

#### 2.5.3 *In vitro* fermentation using a simulated gut model

Forty-five sterile anaerobic tubes were distributed equally among three groups. For the blank control, each tube was prepared with microbial inoculum (2 mL) and basal medium (8 mL). In the positive-control group, 2 mL of microbial inoculum was combined with 8 mL of FOS-supplemented medium to achieve a final concentration of 10 mg/mL. Similarly, the PECD group contained 2 mL of microbial inoculum and 8 mL of PECD-supplemented medium, resulting in the same final concentration. All samples were first mixed thoroughly using a vortex mixer and then incubated at 37 °C under strictly anaerobic conditions maintained with a gas mixture of 85% N_2_, 5% CO_2_, and 10% H_2_. At each of the predetermined sampling times (0, 6, 12, 24, and 48 h), three parallel tubes were withdrawn from every group. Thereafter, the cultured fermentation mixtures were spun down at 2,500 × *g* for 10 min at 4 °C, after which additional measurements were conducted.

#### 2.5.4 Structural characterization analysis of PECD after *in vitro* fermentation

PECD-derived aliquots obtained from the fermentation systems were sampled at predetermined intervals. Total carbohydrate content was determined using the phenol-sulfuric acid procedure, while reducing sugar levels were quantified by the 3,5-dinitrosalicylic acid assay. In addition, molecular weight distribution, monosaccharide composition, and FT-IR spectral characteristics were further examined in accordance with above procedures in simulated digestion.

#### 2.5.5 Profiling of the intestinal microbial community

After 48 h of fermentation, samples from each group were subjected to centrifugation, and the precipitated biomass was harvested for subsequent analysis of the microbial community. Bacterial genomic DNA was extracted from each treatment group using a commercially available DNA isolation kit supplied by Tiangen Biotech (Beijing) Co., Ltd. (Beijing, China). Bacterial 16S rDNA was subjected to amplification of its V3–V4 hypervariable regions, after which sequencing and the accompanying bioinformatic data processing were undertaken by Wuhan Servicebio Biotechnology Co., Ltd. (Wuhan, China). After purification, the amplicons were pooled at equimolar concentrations to construct sequencing libraries with the appropriate adapters. Sequencing of the constructed libraries was performed on an Illumina NovaSeq 6000 platform with a 250 bp paired-end mode. Raw reads were processed using the DADA2 pipeline, which included primer removal, quality filtering, denoising, paired-end merging, and chimera removal. High-quality sequences were resolved into amplicon sequence variants (ASVs), and an ASV abundance table was generated. For taxonomic assignment, ASV representative sequences were classified using the q2-feature-classifier plugin with the classify-sklearn method and a pre-trained Naïve Bayes classifier against the Greengenes database (Release 13.8). The SILVA database (Release 132) was also available as an alternative reference. A confidence threshold of 0.7 was applied for taxonomic assignments; assignments below this threshold were reported as “unclassified” at the corresponding rank. Taxonomy was summarized from domain to species level, with unassigned or uncultured taxa retained at the highest supported level. Based on the ASV dataset, subsequent analyses were performed to evaluate treatment-related alterations in microbial community composition and relative abundance.

#### 2.5.6 SCFAs and metabolomics analysis

At predetermined time points during the *in vitro* fermentation process, PECD samples were collected, and the concentrations of SCFAs were determined using an Agilent 7890A gas chromatography system (Agilent Technologies, Santa Clara, CA, USA) coupled with a flame ionization detector (FID). A flow rate of 20 mL/min was maintained for the carrier gas, which was nitrogen. The detector was maintained at 250 °C, with the respective flow rates of hydrogen, air, and makeup nitrogen adjusted to 40, 400, and 30 mL/min. The injector temperature was maintained at 240 °C, and aliquots of 1 μL were introduced under a split mode of 12:1. The oven temperature for the column was set to 100 °C for the first 0.5 min, followed by a linear rise of 4 °C per minute until reaching 160 °C.

Untargeted metabolomics analysis was performed using a UPLC-Q Exactive HF-X Orbitrap high-resolution mass spectrometry system. Chromatographic separation was achieved on an ACQUITY UPLC HSS T3 column (2.1 mm × 100 mm, 1.8 μm) with a flow rate of 0.4 mL/min, a column temperature of 40 °C, and an injection volume of 2 μL. The mobile phase consisted of 0.1% formic acid in water (A) and 0.1% formic acid in acetonitrile (B), and the gradient elution program was as follows: 0–1.0 min, 5% B; 1.0–4.7 min, 5%−95% B; 4.7–6.0 min, 95% B, 6.0–6.1 min, 95%−5% B; and 6.1–8.5 min, 5% B. The mass spectrometer was equipped with a heated electrospray ionization (HESI) source operating in both positive and negative ion data-dependent acquisition modes. The ion-source parameters were as follows: spray voltage 3.5 kV, sheath-gas flow rate and auxiliary-gas flow rate of 10 and 40 arbitrary units, capillary temperature 320 °C, and auxiliary-gas heater temperature 300 °C. The scan range was set to m/z 70–1,000.

Metabolite annotation was performed using the PerSonalbio Next-Generation Metabolomics Database (PSNGM). This integrated database comprises an in-house spectral library constructed using authentic standards, mzCloud (https://www.mzcloud.org/), LIPID MAPS (https://www.lipidmaps.org/), the Human Metabolome Database (HMDB, https://hmdb.ca/), MassBank of North America (MoNA; https://mona.fiehnlab.ucdavis.edu/), the NIST 2020 MS/MS library, and an artificial intelligence-predicted MS/MS spectral library. Metabolites were annotated by matching the accurate precursor-ion masses and MS/MS fragmentation patterns of the experimental features against those in the reference libraries. MS1 and MS2 matching mass tolerances were 0.01 and 0.05 Da, respectively. Metabolic features with an identification score ≥70 were kept for downstream analysis. Principal component analysis (PCA) serves as a multivariate statistical approach for unsupervised data dimension reduction, can help us extract key information from massive metabolic data, simplify complex metabolic patterns into several principal components, and facilitate the observation of the overall distribution pattern of the sample. Orthogonal partial least squares discriminant analysis (OPLS-DA) represents a supervised statistical modeling technique that can screen metabolic variables highly correlated with specific grouping information, more accurately revealing differences in metabolites between different groups, and conducting in-depth analysis of overall metabolic changes. Metabolites were defined as differential candidates when they fulfilled all of the following criteria: a variable importance in projection (VIP) score > 1.0, an absolute log2 fold change exceeding 1.0, and differences were assessed by an unpaired Student's *t*-test, with statistical significance defined as *p* < 0.05.

### 2.6 Statistical analysis

Each experiment was conducted using no fewer than three independent biological replicates. All data were reported as mean ± SD. Statistical significance among different groups was assessed using one-way analysis of variance (ANOVA), and multiple pairwise comparisons were further conducted with Tukey's honestly significant difference (HSD) *post hoc* test using SPSS Statistics 20.0 software (IBM Corp, Armonk, NY, USA). Differences were regarded as statistically significant when *p* was < 0.05.

## 3 Results

### 3.1 *In vivo* dynamic distribution of FPECD

The distribution of FPECD in mice at specific time points after oral administration was shown in Figure 1. The research results evidenced that the signal intensity of FPECD was predominantly confined to the gastrointestinal tract within the mouse (Figure 1A). The detailed distribution of FPECD was displayed through primary tissue imaging (Figure 1B). From the figure, it can be clearly seen that the fluorescence signal of FPECD was mostly dispersed in the main components of the digestive system, which exhibited strong fluorescence signals. No significant FPECD fluorescence signals were observed in other important organs, including lung, spleen, liver, and kidney tissues. As shown in Figure 1C, the small intestine's signal intensity first increased, peaked at the time point of 1 h, and subsequently showed a downward trend. Meanwhile, the emitted fluorescence-derived signals within the cecal and colonic regions gradually increased over time, reaching their maximum values at approximately 4 h. These results showed that PECD was largely not absorbed and scarcely distributed to the major organs (Liu et al., 2025).

**Figure 1:**
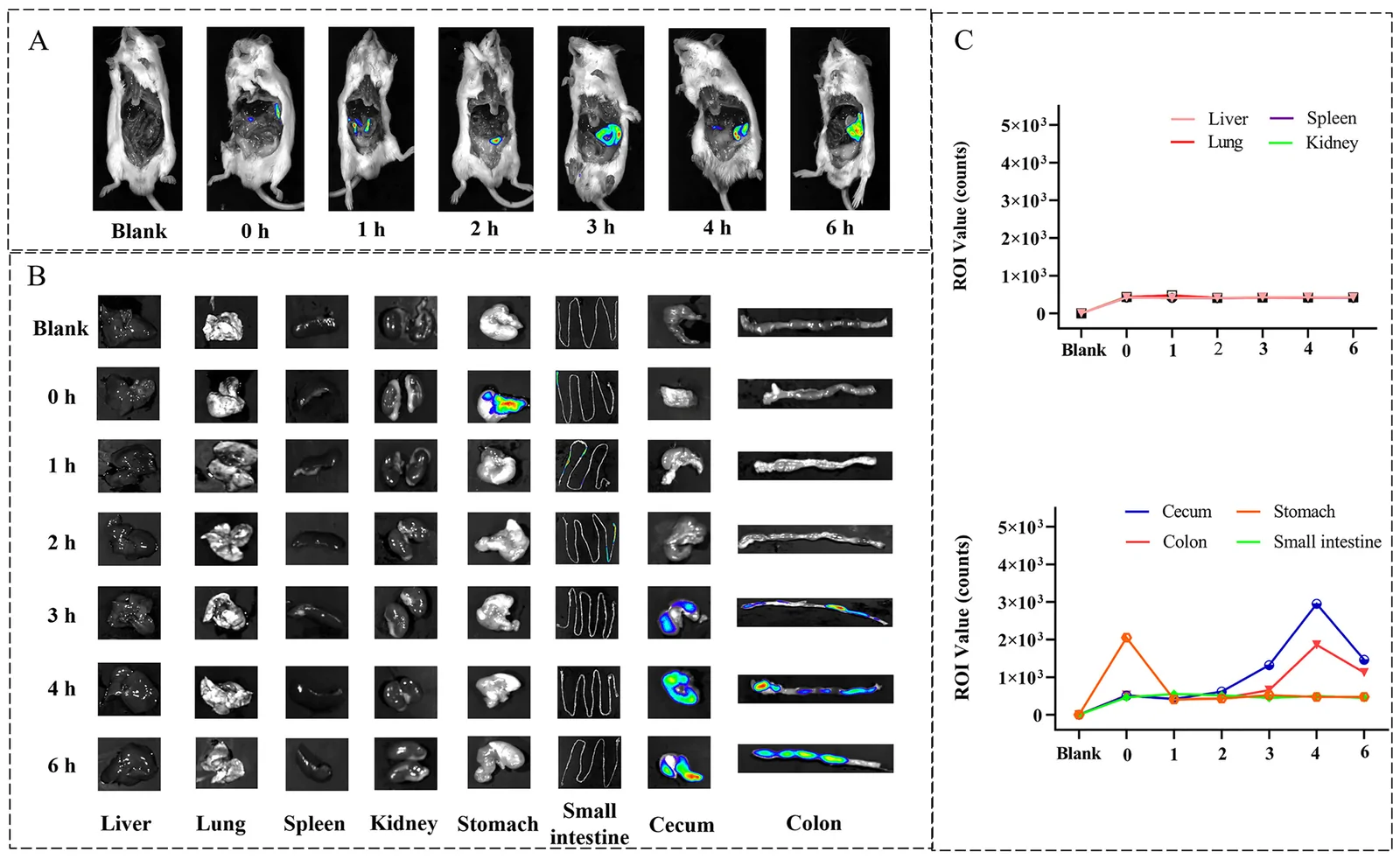
Time-dependent biodistribution of FPECD in mice following oral gavage. **(A)** Representative fluorescence images of whole-body mice at different post-administration time points. **(B)** *Ex vivo* fluorescence imaging of major tissues collected from mice. **(C)** Quantitative analysis of region-of-interest (ROI) signals in different tissues.

### 3.2 *In vitro* simulated digestion results of PECD

#### 3.2.1 Results of the simulated salivary digestion

In human carbohydrate digestion, initial processing occurs in the oral cavity where salivary amylase serves as a critical digestive enzyme, degrading certain starches into glucose or oligosaccharides. Salivary α-amylase mediated carbohydrate digestion by hydrolyzing starch into glucose and oligosaccharides. According to Table 2, total sugar content remained stable during simulated salivary digestion, with no significant change from the initial value of 2,091.4 ± 21.48 to 2,077.42 ± 17.07 μg/mL after digestion (*p* > 0.05). Similarly, the reducing sugar content also exhibited no significant variation throughout the digestion process (from 0.168 ± 0.007 to 0.160 ± 0.033 mg/mL, *p* > 0.05). Consistently, throughout the salivary phase of simulated digestion, the high-performance gel permeation chromatography (HPGPC) chromatograms of PECD exhibited essentially unchanged retention times (Figure 2A), indicating that the molecular-weight distribution of PECD remained relatively stable during salivary digestion. No free monosaccharides were detected (Figure 2D), collectively evidencing PECD's stability against salivary α-amylase. These structural findings are consistent with the initial PECD spectroscopic data and align with the structural and spectroscopic characteristics of polysaccharides reported in previous studies (Cui et al., 2016; Li et al., 2022), thereby supporting the reliability of the present results.

**Table 2:** Changes of total sugar and reducing sugar contents of PECD in simulated digestion process at different time points.

Digestive stage	Digestion time	Total sugar (μg/mL)	Reducing sugar (mg/mL)
Saliva digestion	0 min	2,091.40 ± 21.48 ^a^	0.168 ± 0.007 ^a^
	5 min	2,085.34 ± 21.57 ^a^	0.140 ± 0.038 ^a^
	15 min	2,066.67 ± 15.24 ^a^	0.140 ± 0.026 ^a^
	30 min	2,077.42 ± 17.07 ^a^	0.160 ± 0.033 ^a^
Gastric digestion	0 h	741.94 ± 33.99 ^a^	0.210 ± 0.037 ^a^
	0.5 h	770.00 ± 23.39 ^a^	0.218 ± 0.031 ^a^
	1 h	770.97 ± 28.67 ^a^	0.230 ± 0.007 ^a^
	2 h	722.58 ± 14.78 ^a^	0.218 ± 0.043 ^a^
	4 h	725.81 ± 8.53 ^a^	0.210 ± 0.037 ^a^
	6 h	719.35 ± 14.06 ^a^	0.214 ± 0.028 ^a^
Small intestinal digestion	0 h	459.14 ± 30.66 ^a^	1.619 ± 0.036 ^a^
	0.5 h	457.85 ± 22.90 ^a^	1.553 ± 0.074 ^a^
	1 h	477.42 ± 19.35 ^a^	1.619 ± 0.056 ^a^
	2 h	486.02 ± 15.91 ^a^	1.549 ± 0.091^a^
	4 h	459.35 ± 18.23 ^a^	1.561 ± 0.080 ^a^
	6 h	464.19 ± 16.52 ^a^	1.532 ± 0.075 ^a^

In each column, superscript letters indicate the *post hoc* grouping results for total sugar and reducing sugar during *in vitro* simulated gastrointestinal digestion. Values assigned the same letter are not significantly different (*p* > 0.05), whereas values assigned different letters differ significantly (*p* < 0.05).

**Figure 2:**
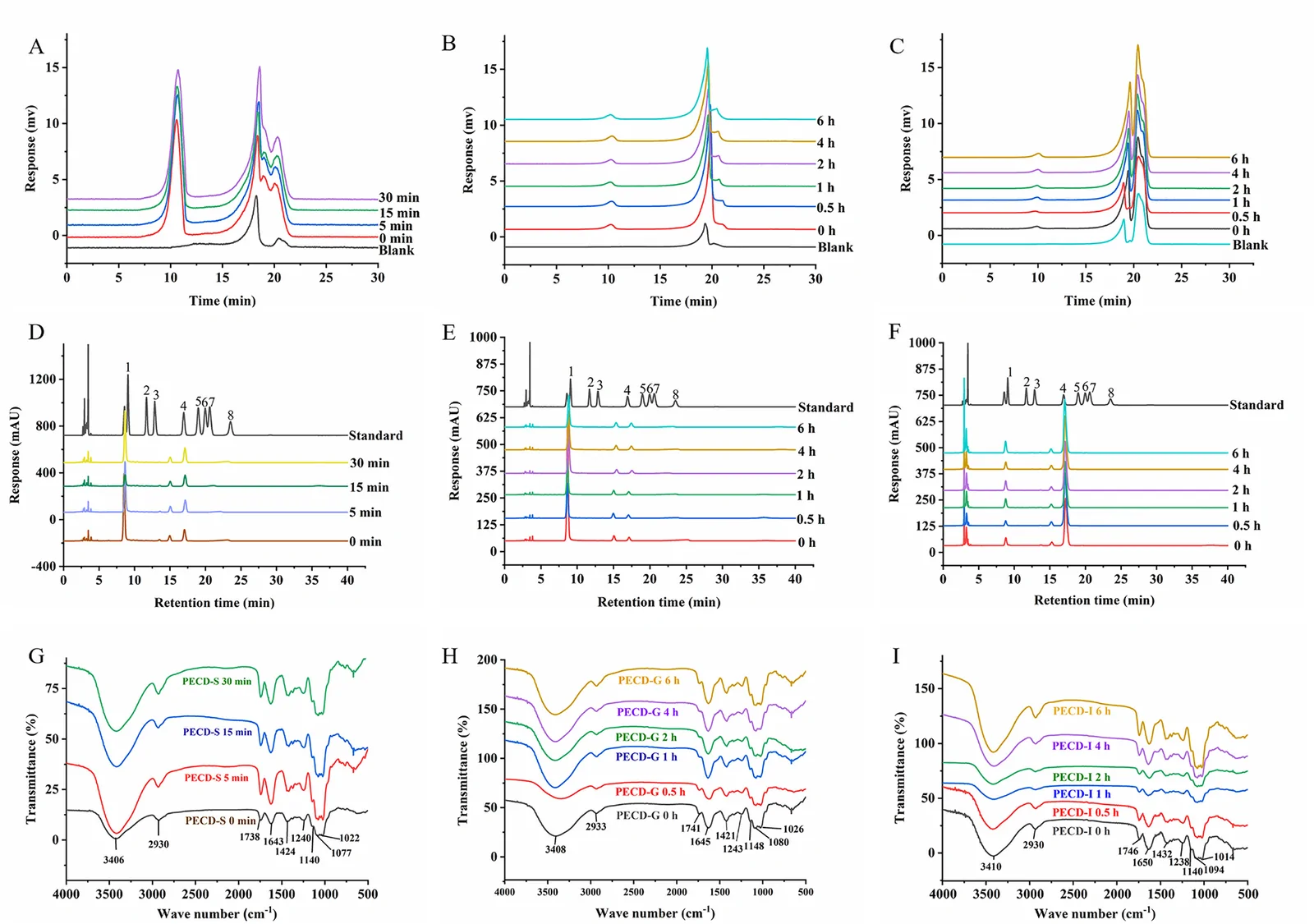
Changes in structural characteristics of PECD during simulated digestion. Molecular weight distribution profiles of PECD during **(A)** simulated salivary digestion, **(B)** simulated gastric digestion, and **(C)** simulated intestinal digestion. Monosaccharide composition profiles during **(D)** salivary digestion, **(E)** gastric digestion, and **(F)** intestinal digestion. Peaks 1–8 correspond to monosaccharide standards of Man, GlcA, GalA, Glc, Xyl, Gal, Ara, and Fuc, respectively. FT-IR spectra during **(G)** salivary digestion, **(H)** gastric digestion, and **(I)** intestinal digestion.

#### 3.2.2 Results of the simulated gastric juice digestion

Investigating the effects of gastric fluid on polysaccharides is fundamental to understanding their structural integrity and functional availability during oral delivery. Figure 2B presented the HPGPC profiles of PECD collected at different time points during simulated gastric digestion. Unlike some polysaccharides reported to degrade during digestion (Li J. et al., 2024) During the gastric phase, the HPGPC elution profile of PECD remained unchanged (Figure 2B), and the results indicated that the molecular weight of PECD remained relatively stable during gastric digestion. This finding was further confirmed by the data summarized in Table 2. During simulated gastric digestion, neither total sugar content (from 741.94 ± 33.99 to 719.35 ± 14.06 μg/mL) nor reducing sugar content (from 0.210 ± 0.037 to 0.214 ± 0.028 mg/mL) exhibited significant variation (all *p* > 0.05). Moreover, no free monosaccharides were detected, as shown in Figure 2E, demonstrating that PECD remained stable and did not undergo obvious degradation in simulated gastric fluid. Overall, these findings suggest that PECD has resistance to gastric digestion, which may be due to its unique structural properties such as compact conformation, specific glycosidic bonds, or resistance to acid hydrolysis.

#### 3.2.3 Results of the simulated small intestinal digestion

The stability of PECD under simulated small intestinal conditions was systematically assessed. From Table 2, during simulated small intestinal digestion, both total sugar content (from 459.14 ± 30.66 to 464.19 ± 16.52 μg/mL) and reducing sugar content (from 1.619 ± 0.036 to 1.532 ± 0.075 mg/mL) remained stable (both *p* > 0.05), indicating PECD's resilience to intestinal environments and pancreatic enzymes. As illustrated in Figure 2C, during the intestinal phase, the HPGPC main peak retention time of PECD remained essentially unchanged, indicating that the molecular weight distribution of PECD was stable under simulated intestinal conditions. Figure 2F further demonstrated the absence of free monosaccharides in PECD-digested intestinal fluid at all time points, where the observed singular peak likely originated from digestive components within the simulated gastrointestinal fluids. Collectively, these results indicate that PECD resists enzymatic degradation by intestinal hydrolases (Luo et al., 2023). These findings indicated that PECD remained intact during its passage through the upper gastrointestinal tract and could reach the large intestine in its natural form. This resistance to digestion makes PECD a potential candidate for prebiotics, as its complete entry into the colon may enable it to serve as a substrate for the gut microbiota, thereby regulating microbial composition and host health.

#### 3.2.4 Changes in FT-IR

As presented in Figures 2G–I, FTIR spectra demonstrated that PECD retained the characteristic absorption bands of polysaccharides throughout the simulated salivary, gastric, and intestinal digestion stages. The broad and intense bands at approximately 3,406–3,410 cm^−1^ were assigned to O-H stretching vibrations, reflecting extensive intra- and intermolecular hydrogen bonding within PECD, whereas the bands at 2,930–2,933 cm^−1^ corresponded to C-H stretching vibrations of the sugar rings and side chains. The absorption bands observed at 1,738–1,746, 1,643–1,650, and 1,421–1,432 cm^−1^ were primarily associated with esterified carbonyl groups, bound water, and carboxyl groups of uronic acid residues, respectively. The region between 1,200 and 1,000 cm^−1^ represented the characteristic fingerprint region of polysaccharides, in which the bands at approximately 1,140–1,148 cm^−1^ were mainly attributed to C-O-C stretching vibrations of glycosidic linkages, while those at 1,077–1,094 and 1,014–1,026 cm^−1^ were predominantly associated with C-O and C-C stretching vibrations within the sugar-ring backbone. Notably, no marked disappearance or substantial shift of the characteristic O-H, C-O, and C-O-C absorption bands, nor the appearance of new characteristic bands, was observed during simulated digestion, indicating that the major functional groups and glycosidic backbone of PECD were largely preserved across the different digestive stages (Wu et al., 2021).

### 3.3 *In vitro* simulated fermentation results of PECD

#### 3.3.1 Change of pH

pH variation was a key parameter for evaluating the fermentation behavior of polysaccharides, with a progressive decrease typically observed due to the continuous generation of acidic metabolites during fermentation (Chen et al., 2018). As can be observed from Figure 3A, the order of initial pH values (>7.0) at 0 h was FOS > blank > PECD, and PECD's lower initial pH was attribute to its uronic acid content. Throughout the 6–48 h period, the blank group maintained significantly elevated pH values relative to both PECD and FOS groups. The PECD fermentation medium exhibited the most rapid pH decline during 0–6 h, stabilizing after 6 h, while FOS demonstrated a consistent downward trend with its steepest decrease likewise occurring within 0–6 h. Specifically, the pH of FOS decreased from 7.23 to 4.41 (ΔpH = 2.82), while that of PECD decreased from 6.87 to 4.63 (ΔpH = 2.24). From 12 to 48 h, the PECD group maintained significantly higher pH values than FOS. The observed decline in the pH of the culture medium was primarily attributed to the gradual increase in SCFAs generated over the course of fermentation.

**Figure 3:**
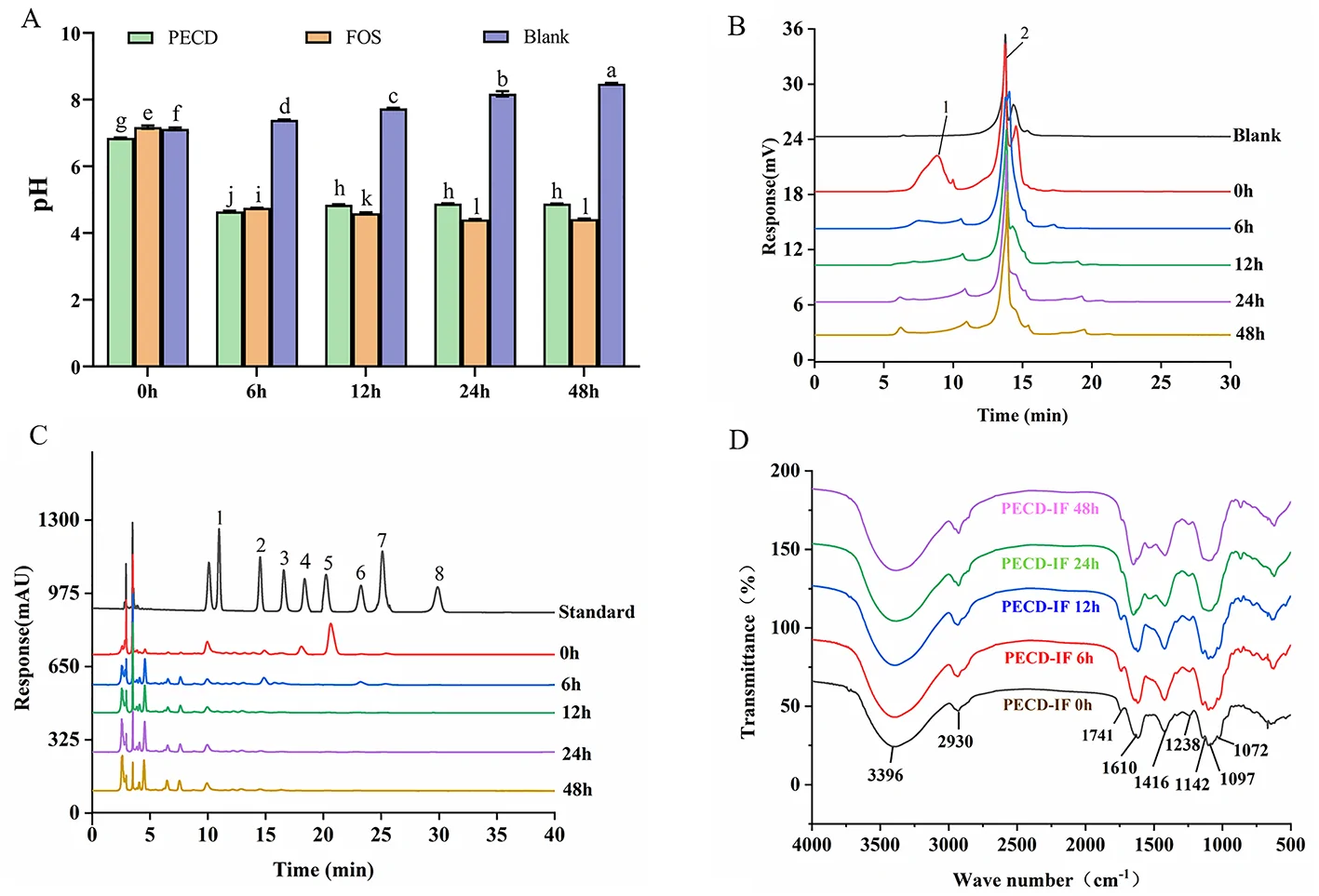
*In vitro* fermentation dynamics. **(A)** pH variation; **(B)** Molecular weight distribution profiles; **(C)** Monosaccharide composition (peaks 1–8 correspond to standards of Man, GlcA, GalA, Glc, Xyl, Gal, Ara, and Fuc, respectively); **(D)** FT-IR spectra of PECD.

#### 3.3.2 Analysis of residual carbohydrate, reducing sugar content, molecular weight and free monosaccharides

With the polymeric carbohydrate content measured in the fermentation medium at 0 h defined as the baseline, the residual proportion at each subsequent stage of fermentation was quantitatively determined. Comprehensive data corresponding to these observations are summarized in Table 3. The residual carbohydrate rates in the FOS group were 73.31 ± 0.51%, 58.16 ± 0.34%, 9.49 ± 0.34%, and 7.46 ± 0.22% at 6, 12, 24, and 48 h of fermentation, respectively. The carbohydrate residue rates in the PECD group were 68.55 ± 1.11%, 42.2 ± 1.00%, 36.42 ± 0.53%, and 33.48 ± 0.5% at 6, 12, 24, and 48 h of fermentation, respectively. As fermentation continued, the fraction of carbohydrates remaining in each group declined significantly (*p* < 0.05). Compared to the initial level at 0 h, the total carbohydrate consumption rates at 48 h reached 92.54% for FOS and 66.52% for PECD. Taken together, the data demonstrated that PECD underwent effective utilization by the gut microbiota throughout the course of *in vitro* fermentation. In the FOS group, substrate utilization decreased continuously during the first 24 h. Although the rate of decline became less pronounced thereafter, a statistically significant difference remained evident between the 24 h and 48 h time points (*p* < 0.05). Moreover, the most rapid decrease in the PECD group occurred during 0–6 h, whereas the steepest decline in the FOS group was detected during 12–24 h. It is noteworthy that, over the 12–48 h *in vitro* fermentation period, the remaining carbohydrate fraction was consistently higher in the PECD group relative to the FOS group. The result indicated that PECD exhibited lower fermentation rates or higher microbial degradation resistance. This can enable polysaccharides to reach the distal colon and maintain long-term release of fermentable substrates, potentially promoting a more balanced and sustained production of beneficial metabolites such as SCFAs (Canfora et al., 2022). In addition, the corresponding changes in reducing sugar content observed during PECD fermentation are presented in Table 3. Specifically, the concentration of reducing sugars decreased progressively from 1.41 ± 0.01 mg/mL at baseline to 1.19 ± 0.02 mg/mL after 6 h (*p* < 0.05), declined further to 0.79 ± 0.01 mg/mL at 12 h (*p* < 0.05), and subsequently remained almost unchanged until 48 h. This pattern indicated that the gut microbiota differed in their capacity to metabolize individual carbohydrate fractions.

**Table 3:** Determination of polysaccharide content during *in vitro* fermentation process.

Fermentation time (h)	Residual carbohydrate (% initial)		Reducing sugar (μg/mL)
	PECD	FOS	
0	100.00 ± 0.00 ^a^	100 ± 0.00 ^a^	1.41 ± 0.01^a^
6	68.55 ± 1.11 ^b^	73.31 ± 0.51 ^b^	1.19 ± 0.02 ^b^
12	42.20 ± 1.00 ^c^	58.16 ± 0.34 ^c^	0.79 ± 0.01 ^c^
24	36.42 ± 0.53 ^d^	9.49 ± 0.34 ^d^	0.76 ± 0.02 ^c^
48	33.48 ± 0.50 ^e^	7.46 ± 0.22 ^e^	0.78 ± 0.01 ^c^

Superscript letters within each column denote the multiple-comparison results for remaining carbohydrate and reducing sugar during the *in vitro* simulated gastrointestinal digestion process. Values sharing the same superscript do not differ significantly (*p* > 0.05), whereas values with different superscripts differ significantly (*p* < 0.05).

#### 3.3.3 Changes in structural characteristics of PECD

The fermentability of natural polysaccharides, as well as their capacity to modulate the gut microbiota, is largely determined by their structural features, elucidating the structural changes of PECD during fecal fermentation is of great significance for revealing its structure-activity relationship (Bang et al., 2018; Wu et al., 2023). Figure 3B illustrated the HPGPC chromatographic profiles of PECD fermentation broth samples collected at different stages of fermentation. Compared with 0 h, the chromatographic peak intensity decreased markedly after 6 h of fermentation. Thereafter, the principal peak progressively shifted to longer retention times at 12 and 24 h, accompanied by evident peak broadening and splitting, indicating gradual cleavage of glycosidic linkages and depolymerization of PECD into lower-molecular-weight fractions. After 48 h, the original high-molecular-weight peak had almost disappeared, further demonstrating extensive microbial degradation of PECD. As presented in Supplementary Table S1, during fermentation, the Mw and Mn of PECD peak 1 decreased significantly over time (both *p* < 0.05), accompanied by notable changes in polydispersity index (PDI). For peak 2, however, Mw and Mn exhibited a biphasic trend, first decreasing and then increasing, while its PDI showed a same change. These distinct molecular weight profiles suggested that peak 1 underwent sustained degradation, whereas peak 2 may represented intermediate degradation products that temporarily accumulated and then further broken down. Collectively, these quantitative data provide strong evidence for the progressive depolymerization of PECD during fermentation, revealing both the overall extent and the differential behavior of its components. Consistently, Figure 3C showed that the characteristic peaks of free Glc, Gal, and Ara vanished after 6 h, whereas the HPLC profiles remained essentially unchanged from 6 to 48 h, indicating that no new free monosaccharides were produced.

As shown in Figure 3D, the overall FT-IR profiles of fecal-fermented PECD remained broadly similar throughout fermentation, suggesting that the core polysaccharide framework was largely retained, whereas specific structural domains underwent selective transformation. Notably, the progressive attenuation of the band at 1,741 cm^−1^, accompanied by increased peak sharpness and its near disappearance at prolonged fermentation times, indicated a gradual loss of esterified carboxyl groups. This phenomenon likely reflects degradation of highly esterified pectin domains (Ma et al., 2020). This interpretation is in agreement with earlier studies demonstrating that pectinolytic gut bacteria selectively degrade pectin substrates with different degrees of esterification and harbor the essential enzymatic machinery required for pectin depolymerization (Calvete-Torre et al., 2024; Steigerwald et al., 2024).

#### 3.3.4 Changes in microbiome composition during *in vitro* fermentation

Compared with the Blank 0 h group, the Blank 48 h group exhibited significantly lower Chao1 (1,047.15 ± 199.77 vs. 569.12 ± 36.00, *p* < 0.01) and Shannon indices (7.174 ± 0.113 vs. 6.052 ± 0.098, *p* < 0.001), whereas the Simpson index showed no significant difference between the two groups (*p* > 0.05) (Figures 4A–C). These findings indicated that prolonged static fermentation in the absence of an exogenous carbohydrate substrate led to a reduction in microbial richness and community evenness. This background decline may be attributable to progressive nutrient depletion, selective survival of microorganisms capable of utilizing endogenous substrates, and the accumulation of fermentation end-products. Similar reductions in microbial richness and evenness have been reported in substrate-limited batch fermentation systems (Fu et al., 2025; Wongsanittayarak et al., 2025). No statistically significant difference in the Chao1 index was detected between the FOS 48 h and Blank 48 h groups (614.08 ± 35.73 vs. 569.12 ± 36.00, *p* > 0.05). However, compared with the Blank 48 h group, both the Shannon and Simpson indices of the FOS 48 h group further decreased, with the Shannon index dropping from 6.052 ± 0.098 to 5.541 ± 0.187 (*p* < 0.05) and the Simpson index decreasing from 0.966 ± 0.002 to 0.944 ± 0.002 (*p* < 0.05), suggesting that FOS was selectively consumed by certain rapidly fermenting saccharolytic taxa, thereby altering community evenness rather than expanding overall richness (Mahalak et al., 2022). Notably, the PECD 48 h group displayed the lowest values across all three alpha-diversity indices and was significantly lower than the Blank 48 h group, with the Chao1 index decreasing from 569.12 ± 36.00 to 281.06 ± 29.34 (*p* < 0.05), the Shannon index declining from 6.052 ± 0.098 to 3.811 ± 0.233 (*p* < 0.001), and the Simpson index dropping from 0.966 ± 0.002 to 0.732 ± 0.016 (*p* < 0.001). This phenomenon is commonly observed during the microbial fermentation of fermentable polysaccharides and may result from the selective proliferation of substrate-responsive taxa, which increases their competitive dominance and consequently reduces community evenness and overall α-diversity (Xiao et al., 2022). These findings suggest that, under the present fermentation conditions, PECD may exert a prebiotic-like effect primarily through substrate-specific enrichment of responsive microorganisms and the consequent ecological restructuring of the microbial community, rather than through a generalized increase in alpha diversity (Ge et al., 2024; Popov et al., 2024; Hutkins et al., 2025). However, these correlative observations require functional validation in disease models to confirm causality. Sparse curves plateaued with increased sequencing depth, confirming data reliability (Figure 4E). Observed operational taxonomic unit (OTU) counts corroborated the species richness decline, consistent with Chao1 trends. Venn analysis demonstrated inter-group taxonomic disparities (Figure 4D), indicating microbiota compositional shifted necessitating further analysis. In hierarchical clustering analysis, the clustering of duplicate samples within the same group is good, indicating reliable repeatability within the group. There is clear branching separation between different treatment groups, with PECD 48 h samples clustered into independent branches, indicating the most prominent reshaping of community structure (Figure 4F). PCoA provided additional support for this conclusion, with the first and second coordinate axes explaining 54.3% and 23.8% of the total variation in community structure, respectively, with a total explanatory power of 78.1% (Figure 4G). The samples of each group were clearly separated in the coordinate space, with PECD 48 h being the farthest from the other groups, indicating that it induced the strongest community migration. There was also a distinguishable structural shift between FOS 48 h and Blank 48 h groups, but the overall amplitude was lower than PECD 48 h.

**Figure 4:**
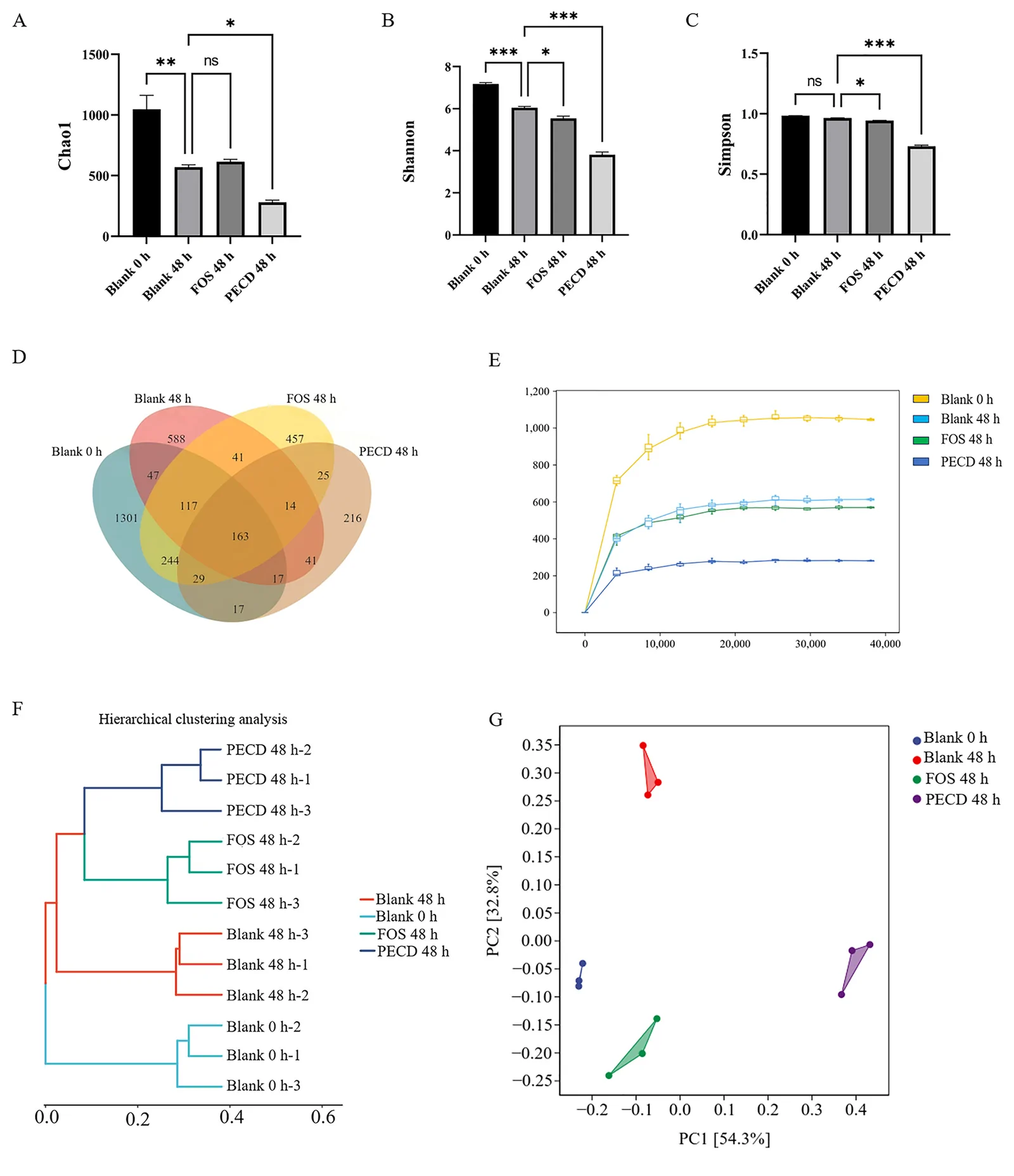
Shifts in the gut microbial community during *in vitro* fermentation of PECD. **(A)** Chao1 richness estimator; **(B)** Shannon diversity index; **(C)** Simpson diversity index; **(D)** Venn plot; **(E)** Rarefaction analysis; **(F)** Hierarchical clustering dendrogram based on Bray–Curtis distances; **(G)** PCA analysis of gut microbiota structure in different samples. Statistical significance is denoted as follows: ^*^p < 0.05, ^**^p < 0.01, ^***^p < 0.001, and ns, not significant.

As illustrated in Figure 5A, in every group, the composition of gut microbiota was primarily characterized by the dominance of the phyla *Bacillota, Bacteroidota, Pseudomonadota*, and *Actinomycetota*. Following 48 h of *in vitro* fermentation, the relative abundances of *Bacillota* and *Bacteroidota* decreased significantly in the Blank 48 h group compared with the Blank 0 h group, from 59.73 ± 6.52% to 13.73 ± 1.20% and from 31.64 ± 6.14% to 4.84 ± 1.34%, respectively (both *p* < 0.001). Compared with the Blank 48 h group, the FOS 48 h group markedly increases in these two phyla, reaching 52.83 ± 9.48% (*p* < 0.001) and 13.73 ± 1.19% (*p* < 0.05), respectively, whereas the PECD group showed no significant changes, with values of 16.41 ± 7.59% and 2.40 ± 0.95%, respectively (both *p* > 0.05). Relative to the Blank 0 h group, the abundance of *Pseudomonadota* increased notably in the Blank 48 h group (from 5.69 ± 0.82% to 59.56 ± 5.06%, *p* < 0.001). This elevation was significantly suppressed in the FOS 48 h group (19.21 ± 3.26%, *p* < 0.001), while the PECD 48 h group exhibited no obvious difference in *Pseudomonadota* abundance compared with the Blank 48 h group (70.64 ± 8.09% vs. 59.56 ± 5.06%, *p* > 0.05). These results indicated that PECD may have a more selective modulatory effect at the phylum level compared with FOS. In addition, the abundance of *Actinomycetota* tended to decline in the Blank 48 h group vs. the Blank 0 h group (from 2.15 ± 0.09% to 0.88 ± 0.09%, *p* > 0.05). By contrast, both the FOS (13.66 ± 6.07%, *p* < 0.01) and PECD (10.28 ± 2.54%, *p* < 0.05) groups displayed a significant upregulation in the abundance of *Actinomycetota* following 48 h fermentation.

**Figure 5:**
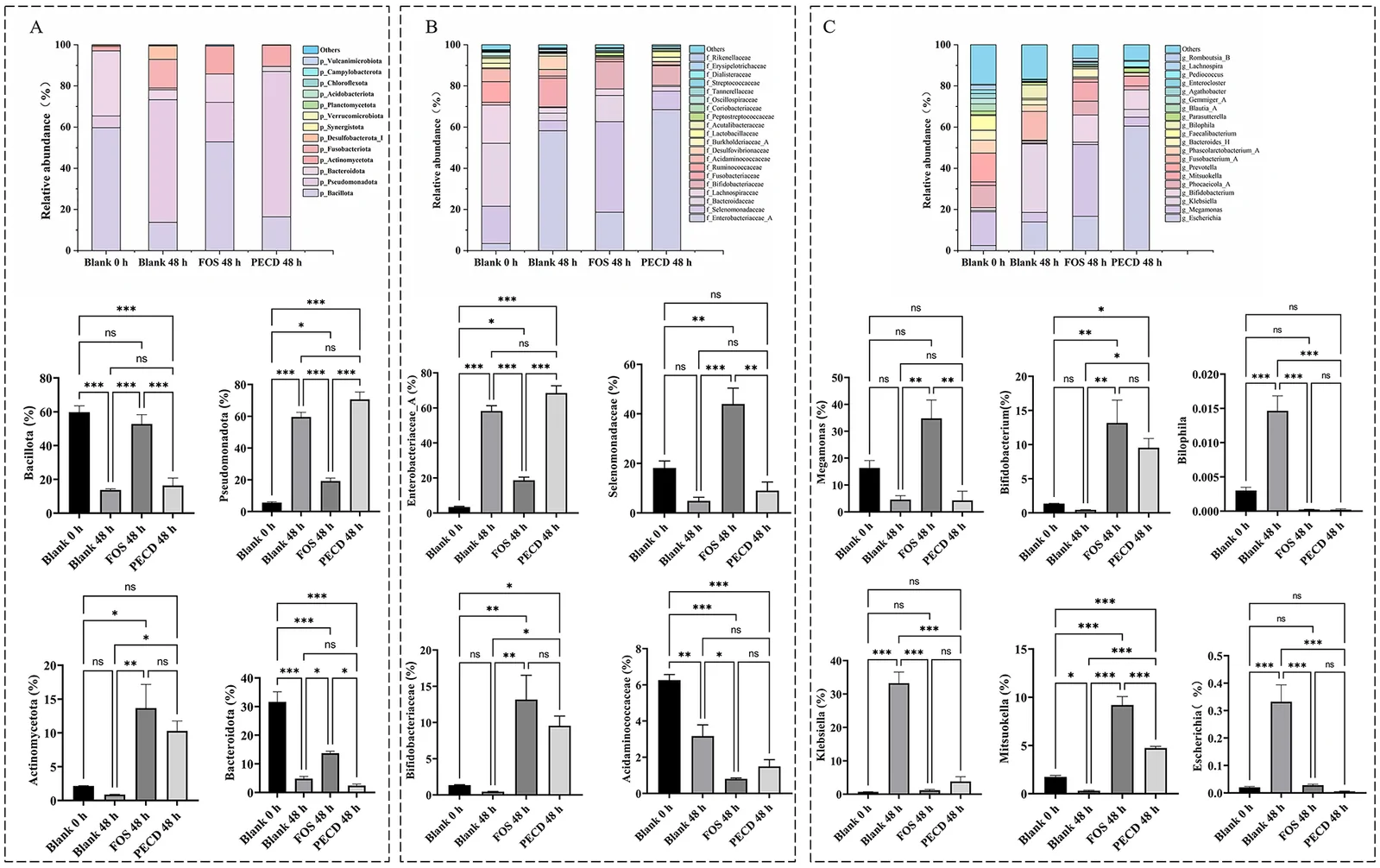
Gut microbiota compositional profiles. **(A)** Proportional taxonomic abundance at the phylum rank; **(B)** Proportional taxonomic abundance at the family rank; **(C)** Proportional taxonomic abundance at the genus rank. Statistical significance is denoted as follows: *** *p* < 0.001, ** *p* < 0.01, * *p* < 0.05, and ns, not significant.

Members of *Bifidobacteriaceae* generally exert beneficial effects on the host by maintaining gut ecological homeostasis, modulating immune responses, and improving bowel function and metabolic outcomes (Li et al., 2023; Takeda et al., 2023). Family-level analysis (Figure 5B) revealed that, relative to the Blank 48 h group (0.44 ± 0.06%), both the FOS 48 (13.17 ± 5.80%, *p* < 0.01) and PECD 48 (9.53 ± 2.34%, *p* < 0.05) groups tended to exhibit significantly higher relative abundance of *Bifidobacteriaceae*, implying that PECD, like FOS, may also modulate this family. By contrast, members of *Selenomonadaceae* appear to be more context-dependent. Some gut taxa participate in dietary substrate fermentation and propionate production, while certain members may also promote host lipid absorption and obesogenic metabolism under specific conditions (Vos et al., 2024). Compared with the Blank 48 h group (4.89 ± 2.51%), the FOS 48 h group showed a significant increase in the abundance of *Selenomonadaceae* to 44.02 ± 11.02% (*p* < 0.001), whereas the PECD 48 h group (9.01 ± 6.03%) exhibited no significant difference (*p* > 0.05). This divergence suggests that, unlike FOS, PECD does not stimulate *Selenomonadaceae*, which might be advantageous if certain members of this family are linked to unfavorable metabolic outcomes. Furthermore, relative to the Blank 48 h group (58.26 ± 5.22%), the *Enterobacteriaceae_A* abundance in the FOS 48 h group decreased significantly to 18.64 ± 3.25% (*p* < 0.001), confirming the well-established inhibitory effect of FOS on this family. However, no significant inhibition was observed in the PECD 48 h group (68.46 ± 7.24%, *p* > 0.05). The lack of suppression may be attributed to differences in fermentation kinetics, metabolite profiles (e.g., lactate or SCFAs production), or substrate specificity between PECD and FOS. Regarding *Acidaminococcaceae*, after 48 h of fermentation, its abundance in the Blank (3.17 ± 1.08%, *p* < 0.01), FOS (0.80 ± 0.08%, *p* < 0.001), and PECD (1.49 ± 0.64%, *p* < 0.001) groups was significantly lower than that in the Blank 0 h group (6.26 ± 0.53%). Moreover, the abundance in the FOS 48 h group was significantly lower than that in the Blank 48 h group (*p* < 0.05), whereas the PECD 48 h group showed only a numerical decrease without statistical significance (*p* > 0.05). Since certain *Acidaminococcus* species are associated with protein fermentation and the production of potentially harmful metabolites (Cornejo-Pareja et al., 2024), their additional reduction by PECD may confer a further health benefit, similar to FOS.

Genus-level analysis (Figure 5C) indicated that, relative to the Blank 48 h group, both the FOS 48 h and PECD 48 h groups exhibited increased abundances of *Bifidobacterium* and *Mitsuokella*, while inhibited the abundance of *Klebsiella*. Specifically, the relative abundance of *Bifidobacterium* increased from 0.44 ± 0.06% in the Blank 48 h group to 13.17 ± 5.80% (*p* < 0.01) and 9.54 ± 2.34% (*p* < 0.05) in the FOS 48 h and PECD 48 h groups, respectively. Previous evidence showed that *Bifidobacterium* was associated with the preservation of intestinal barrier integrity, the enhancement of regulatory T-cell responses, and the modulation of dendritic-cell and macrophage functions, and the attenuation of inflammatory responses. Thus, the enrichment of *Bifidobacterium* by PECD is consistent with a potential bifidogenic effect; however, this compositional change alone is insufficient to demonstrate a host-level health benefit (Gavzy et al., 2023). The observation that PECD, like FOS, significantly promoted *Bifidobacterium* further supported its prebiotic-like potential at the genus level. *Mitsuokella* species have been shown to efficiently degrade phytate and, in concert with *Anaerostipes rhamnosivorans*, to promote propionate production. These metabolic products have also been linked to improved intestinal epithelial barrier integrity (Vos et al., 2024). Compared with the Blank 48 h group (0.30 ± 0.07%), the relative abundance of *Mitsuokella* increased markedly to 9.20 ± 0.87% and 4.74 ± 0.17% in the FOS 48 h and PECD 48 h groups, respectively (both *p* < 0.001). The parallel increase of *Mitsuokella* by both FOS and PECD suggests that PECD may also indirectly support gut barrier function through propionate-related pathways, although direct measurement of propionate would be required for confirmation. Conversely, *Klebsiella* has been significantly associated with disease exacerbation and severity, can aggravate intestinal inflammation in animal models, and may suppress competing gut commensals, thereby enhancing its colonization advantage and reshaping gut microbial ecology (Federici et al., 2022; Tan et al., 2024). In this study, the relative abundance of *Klebsiella* decreased from 33.23 ± 5.80% in the Blank 48 h group to 1.20 ± 0.41% and 3.81 ± 2.46% in the FOS 48 h and PECD 48 h groups, respectively (both *p* < 0.001). The suppression of *Klebsiella* by both FOS and PECD is therefore a favorable outcome. Notably, despite the lack of significant inhibition on the family *Enterobacteriaceae_A* (which includes *Klebsiella*) by PECD as noted in the family-level analysis, the genus-level data indicate that PECD still effectively reduces *Klebsiella* specifically. This discrepancy highlights the importance of genus- and species-level resolution that PECD may selectively suppress certain opportunistic genera within *Enterobacteriaceae* without affecting the entire family. PECD also reduced the relative abundance of *Bilophila*, decreasing from 0.0147 ± 0.0022% in the Blank 48 h group to 0.00022 ± 0.00012% in the PECD 48 h group (*p* < 0.001), with a similarly low abundance observed in the FOS 48 h group (0.00024 ± 0.00004%, *p* < 0.001). The best-characterized species, *Bilophila wadsworthia*, is a bile-tolerant, sulfite-reducing anaerobe capable of utilizing taurine-derived sulfur compounds. Under conditions enriched in taurine-conjugated bile acids, *B. wadsworthia* can expand and promote colitis in genetically susceptible mice, and has been shown to aggravate intestinal barrier dysfunction, inflammation, bile acid dysmetabolism, and metabolic disturbances in high-fat-diet models. Therefore, the decrease in *Bilophila* may indicate a reduction in a sulfidogenic and pathobiont-associated ecological feature (Devkota et al., 2012; Natividad et al., 2018). In contrast, PECD significantly increased the relative abundance of *Escherichia*. This genus is functionally heterogeneous and includes both normal intestinal commensals and strains with pathobiont or pathogenic potential. In the inflamed intestine, host-derived nitrate can confer a respiratory growth advantage on *Escherichia* coli, thereby facilitating its expansion. However, the present *in vitro* fermentation system lacks the complete host inflammatory environment. The observed increase in *Escherichia* may therefore reflect preferential substrate utilization, rapid growth of facultative anaerobes, microbial competition, or compositional redistribution caused by decreases in other taxa rather than the expansion of pathogenic strains (Winter et al., 2013; Kittana et al., 2018).

Unlike FOS, PECD did not significantly enrich *Megamonas* or *Selenomonadaceae_A*, highlighting distinct substrate-selective fermentation profiles. For *Megamonas*, its abundance increased from 4.58 ± 2.48% (Blank 48 h group) to 34.80 ± 11.89% in the FOS 48 h group (*p* < 0.01), whereas no significant change was observed in the PECD 48 h group (4.27 ± 5.94%, *p* > 0.05). Recent studies have shown that fructan chain length and degree of polymerization can determine which microbial populations respond to inulin, FOS, and their degradation products, with only partially overlapping responsive taxa (Riva et al., 2023). FOS has also been reported to promote the growth of *Megamonas* during *in vitro* fecal fermentation (Dhamaratana et al., 2025). However, the physiological significance of *Megamonas* enrichment appears to be species- and context-dependent. Inulin-enriched *M. funiformis* increased intestinal propionate and ameliorated metabolic dysfunction-associated fatty liver disease in experimental models, whereas *M. rupellensis* promoted intestinal lipid absorption and obesity through myo-inositol degradation (Yang et al., 2023; Wu et al., 2024). Given the taxonomic heterogeneity of *Selenomonadaceae*, the absence of enrichment by PECD should therefore not be interpreted as intrinsically beneficial or detrimental. Rather, it likely reflects a structure-dependent fermentation profile distinct from that of FOS, and its functional significance requires targeted SCFA quantification, absolute microbial enumeration, and species- or strain-resolved analysis. Collectively, PECD and FOS imposed distinct substrate-selective pressures on the microbial community. The enrichment of *Bifidobacterium* and *Mitsuokella*, together with reductions in *Klebsiella* and *Bilophila*, supports the selective microbiota-modulating potential of PECD.

Linear discriminant analysis (Figure 6) identified statistically significant microbial biomarkers [linear discriminant analysis (LDA) score > 2] distinguishing sample groups. *Bacillota_A, Bacteroidia* and *Clostridia* were predominantly enriched in Blank 0 h group. Blank 48 h group exhibited primary enrichment of *Klebsiella, Fusobacteriia* and *Fusobacteriales*. The FOS 48 h group demonstrated significant enrichment of *Selenomonadaceae, Bacillota* and *Negativicutes*. PECD 48 h group showed characteristic enrichment of *Pseudomonadota, Enterobacterales_A, Gammaproteobacteria*.

**Figure 6:**
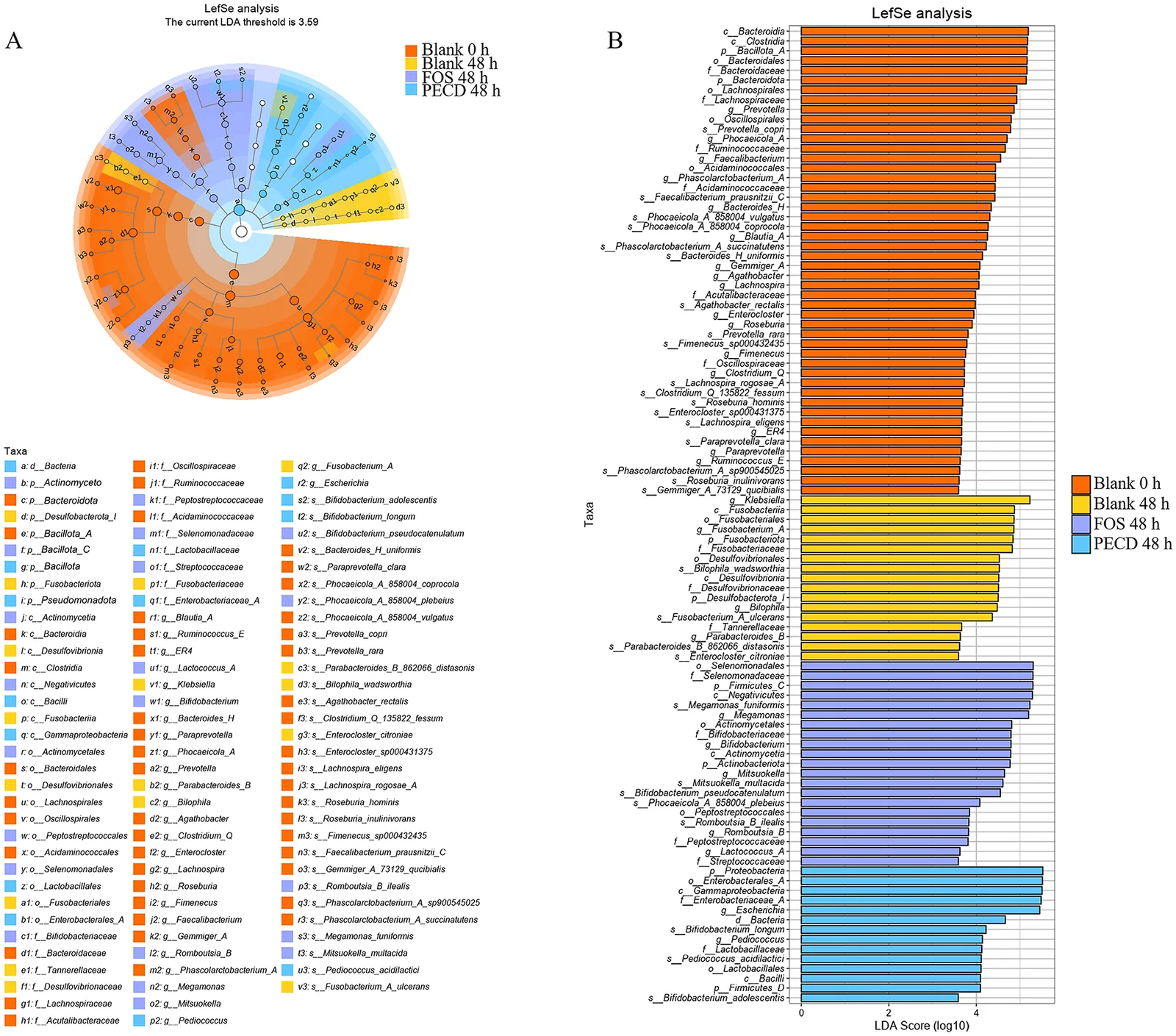
LEfSe-based differential microbial analysis (LDA score > 2). **(A)** OTU-level comparison among Blank 0, Blank 48, FOS 48, and PECD 48 groups; **(B)** LDA score histogram visualization. Blank 0 h: blank medium at 0 h fermentation; Blank 48 h, FOS 48 h, PECD 48 h: blank, FOS, and PECD media following 48-h fermentation.

#### 3.3.5 Changes in SCFAs during *in vitro* fermentation

As presented in Table 4, the concentrations of total SCFAs showed distinct variation patterns among the groups during the 48-h *in vitro* fermentation period. In the Blank group, the total SCFAs concentration significantly decreased after 48 h (*p* < 0.05), dropping from 11.46 ± 1.43 mmol/L at 0 h to 7.34 ± 0.36 mmol/L. FOS exhibited extremely high fermentation utilization efficiency. The total concentration of SCFAs rapidly increased during the fermentation and reached its peak at 24 h (51.75±2.82 mmol/L), then remained unchanged (51.18 ± 2.61 mmol/L, *p* > 0.05) at 48 h. Compared with 0 h, the SCFAs production at 24 h increased by about 3.7 times, confirming that FOS can be rapidly and efficiently fermented by gut microbiota. Unlike the explosive acid production of FOS, the PECD group exhibited a sustained and stable acid production pattern. Although its initial concentration (0 h, 22.63 ± 5.84 mmol/L) was relatively high, it showed a steady increasing trend during the fermentation process and reached its highest value (34.09 ± 1.44 mmol/L) at the end of the fermentation. After 48 h of fermentation, the levels of acetic acid, propionic acid, butyric acid, and valeric acid in both the FOS and PECD groups were significantly higher than those in the Blank group (all *p* < 0.05), with particularly pronounced increases in acetic acid (6.4- and 4.0-fold in FOS and PECD groups, respectively) and butyric acid (13.8- and 8.6-fold, respectively). These results demonstrated the fermentability of both substrates, which is suggestive of prebiotic-like potential. However, the temporal dynamics of SCFA production differed markedly between the two groups. In the PECD group, acetic acid levels showed an upward trend during the initial 0–6 h of fermentation (from 8.80 ± 2.14 to 9.55 ± 0.65 mmol/L), then decreased significantly at 12 h (7.25 ± 0.60 mmol/L, *p* < 0.05) and remained almost unchanged until 48 h (*p* > 0.05). By contrast, acetic acid levels in the FOS group exhibited a continuous upward trend over the entire 48 h (from 5.98 ± 0.41 to 12.38 ± 0.96 mmol/L) and were significantly higher than those in the PECD group between 12 and 48 h (*p* < 0.05). Regarding butyrate, the PECD group showed a slight decrease at 6 h (from 11.95±5.14 to 8.49 ± 0.30 mmol/L) followed by a significant increase thereafter, reaching 20.87 ± 0.90 mmol/L at 48 h. The FOS group displayed a significant increase from 0 to 48 h, with much higher butyrate levels than the PECD group between 12 and 48 h (*p* < 0.05). These distinct kinetic profiles likely reflect differences in fermentation patterns and substrate utilization. FOS, as a rapidly fermentable prebiotic, is efficiently metabolized by a broad range of saccharolytic bacteria (Cui et al., 2022), resulting in sustained and high SCFA production throughout the 48 h period. However, FOS is fermented too fast in the intestine, which is prone to lead to excessive accumulation of gas and SCFAs, thus causing symptoms such as abdominal distension and proximal colon intolerance (Cheng et al., 2025). In contrast, PECD, as a complex polysaccharide mixture derived from ECD, appeared to undergo biphasic fermentation kinetics and a moderate SCFA profile during the fermentation. Despite lower concentrations than FOS, PECD consistently raised SCFA levels above baseline, indicating meaningful activity. These dynamics align with PECD's unique microbiota modulation, for example, enriching *Bifidobacterium* and *Mitsuokella* without stimulating *Selenomonadaceae* or *Megamonas*. Thus, PECD may exhibit a more selective and moderate fermentation pattern compared with FOS. However, this fermentation feature, derived from batch cultures, remained correlative, and *in vivo* validation is required to assess its physiological relevance.

**Table 4:** Changes in SCFAs content in different groups during different time periods of *in vitro* simulated fermentation.

Groups	Fermentation time (h)	SCFAs (mmol/L)				
		Acetic acid	Propionic acid	n-butyric acid	n-valeric acid	Total SCFAs
Blank	0	2.94 ± 0.46 ^b, C^	2.89 ± 0.77 ^a, A^	3.27 ± 0.29 ^a, B^	2.35 ± 0.65 ^a, A^	11.46 ± 1.43 ^a, B^
	6	3.04 ± 0.08 ^b, B^	2.31 ± 0.06 ^ab, B^	2.96 ± 0.17 ^a, B^	2.11 ± 0.31 ^a, A^	10.42 ± 0.52 ^a, B^
	12	3.37 ± 0.18 ^ab, C^	2.29 ± 0.13 ^ab, A^	3.09 ± 0.07 ^a, C^	1.99 ± 0.10 ^ab, B^	10.75 ± 0.41 ^a, C^
	24	3.60 ± 0.09 ^a, C^	2.13 ± 0.08 ^bc, B^	3.19 ± 0.16 ^a, C^	2.00 ± 0.03 ^ab, B^	10.91 ± 0.11 ^a, C^
	48	1.95 ± 0.13 ^c, C^	1.51 ± 0.09 ^c, B^	2.44 ± 0.06 ^b, C^	1.45 ± 0.18 ^b, C^	7.34 ± 0.36 ^b, C^
FOS	0	5.98 ± 0.41 ^d, B^	2.22 ± 0.10 ^b, A^	4.97 ± 0.30 ^d, AB^	0.77 ± 0.05 ^d, B^	14.33 ± 0.92 ^d, B^
	6	10.97 ± 0.84 ^bc, A^	3.29 ± 0.07 ^a, A^	7.97 ± 1.29 ^c, A^	1.14 ± 0.15 ^c, B^	24.13 ± 2.01 ^c, A^
	12	10.65 ± 0.55 ^c, A^	2.46 ± 0.39 ^b, A^	23.34 ± 2.17 ^b, A^	2.49 ± 0.07 ^a, A^	38.23 ± 2.08 ^b, A^
	24	13.60 ± 1.42 ^a, A^	2.16 ± 0.26 ^b, B^	33.76 ± 1.87 ^a, A^	2.08 ± 0.18 ^b, B^	51.75 ± 2.82 ^a, A^
	48	12.38 ± 0.96 ^ab, A^	2.14 ± 0.68 ^b, AB^	33.78 ± 1.46 ^a, A^	2.35 ± 0.15^ab, B^	51.18 ± 2.61 ^a, A^
PECD	0	8.80 ± 2.14 ^ab, A^	2.10 ± 0.15 ^b, A^	11.95 ± 5.14 ^cd, A^	1.35 ± 0.38 ^c, AB^	22.63 ± 5.84 ^c, A^
	6	9.55 ± 0.65 ^a, A^	3.48 ± 0.56 ^a, A^	8.49 ± 0.30 ^d, A^	1.48 ± 0.06 ^c, B^	22.67 ± 1.33 ^c, A^
	12	7.25 ± 0.60 ^b, B^	2.06 ± 0.39 ^b, A^	14.68 ± 0.65 ^bc, B^	2.77 ± 0.22 ^b, A^	26.48 ± 0.70 ^bc, B^
	24	7.63 ± 0.23 ^ab, B^	2.92 ± 0.17 ^a, A^	18.82 ± 0.45 ^ab, B^	3.66 ± 0.14 ^a, A^	31.45 ± 0.78 ^ab, B^
	48	7.85 ± 0.57 ^ab, B^	3.02 ± 0.06 ^a, A^	20.87 ± 0.90^a, B^	3.83 ± 0.18 ^a, A^	34.09 ± 1.44 ^a, B^

Different lowercase letters indicate significant differences (*p* < 0.05) among different time points within the same group, while different uppercase letters denote significant differences (*p* < 0.05) among different groups at the same time point.

#### 3.3.6 Untargeted metabolomic analysis

Metabolites generated by intestinal microorganisms serve as pivotal signaling intermediates in host–microbe crosstalk, enabling two-way interactions between the microbial community and the host and thereby substantially shaping overall host health. As emphasized by Liu et al., metabolomics constitutes a powerful and indispensable analytical strategy for deciphering the intricate, multi-layered interplay between the gut microbiota and the host (Liu W. et al., 2024). This study demonstrated that PECD contributed to changes in the composition of the intestinal microbiota. To further clarify the mechanisms through which PECD affected the gut microbial community, a comprehensive LC-MS/MS-based metabolomic analysis of the culture broth was performed. As shown in Figure 7, PECD markedly influenced the metabolic activity of the intestinal microbiota. PCA further revealed an obvious distinction between the Blank and PECD groups across the two ionization modes (Figures 7A, B), implying that PECD intervention induced marked changes in the metabolic characteristics of the gut microbiota. OPLS-DA was subsequently employed to further assess intergroup differences (Figures 7C–F). Model performance was evaluated using *R*^2^*Y* (goodness of fit) and *Q*^2^ (predictive ability), with *Q*^2^ > 0.5 considered indicative of satisfactory predictive capability. In the positive-ion mode, the OPLS-DA model yielded *R*^2^*Y* and *Q*^2^ values of 1.000 and 0.922, respectively. In the negative-ion mode, the corresponding values were 1.000 and 0.938. To assess the risk of overfitting, a particular concern for supervised multivariate models, especially with limited sample sizes (*n* = 3), permutation tests were performed, yielding *Q*^2^ intercepts of 0.35 and 0.30 for the positive- and negative-ion modes, respectively. While these intercepts suggest some degree of overfitting, likely attributable to the small sample size inherent to the *in vitro* fermentation setup, the overall model performance supports a distinct metabolic separation between the two groups. Moreover, the observed separation was consistent with targeted SCFA quantification, which showed significantly higher levels of acetate, propionate, and butyrate in the PECD group (*p* < 0.05). Nevertheless, given the limitations of the current model, the discriminant metabolites identified herein should be interpreted as exploratory and warrant validation in future studies with larger cohorts. Collectively, these findings confirm significant differences in the metabolite profiles of fermentation samples between the Blank and PECD groups.

**Figure 7:**
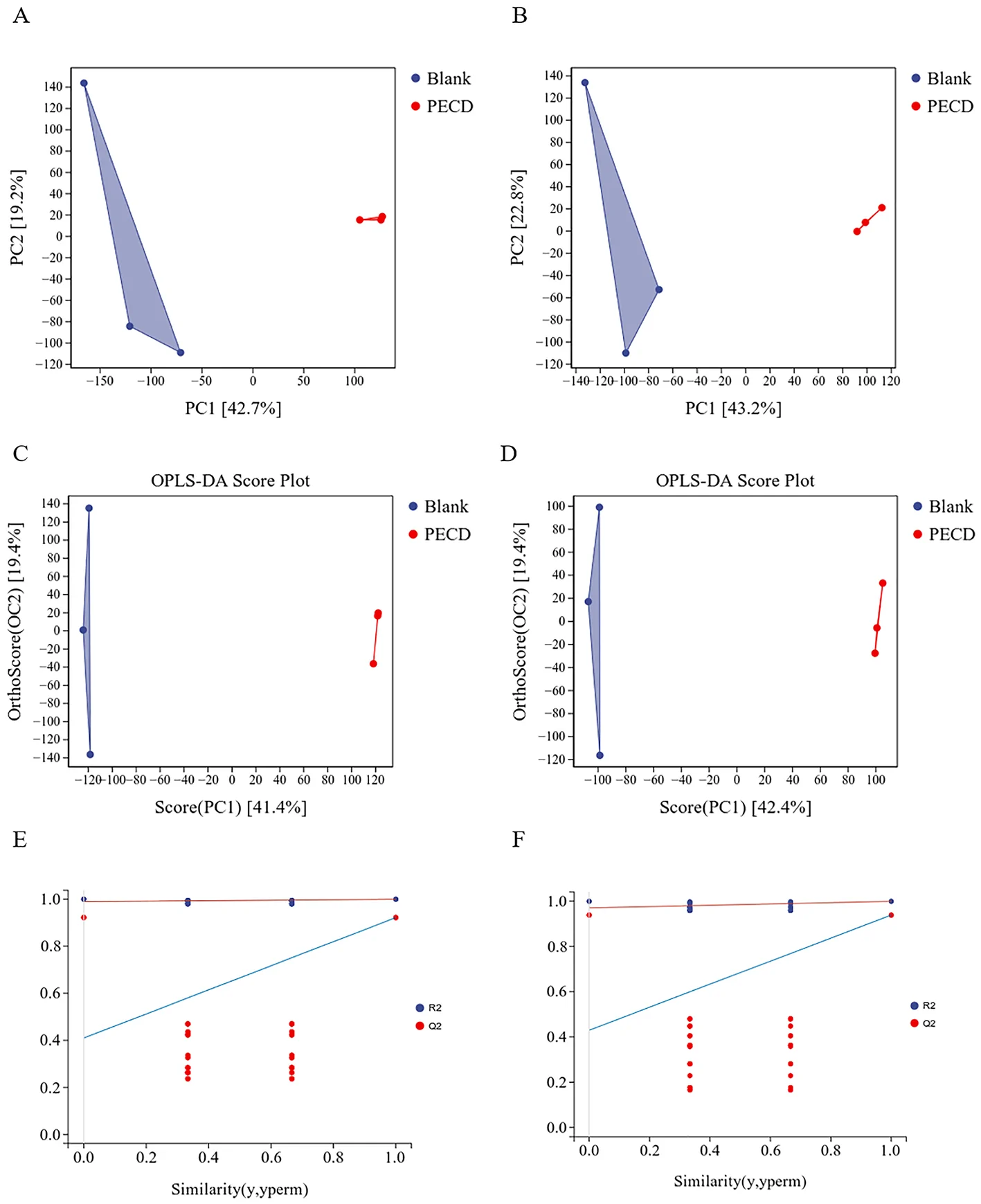
Impact of PECD on metabolites derived from the gut microbiota. **(A)** PCA score plot acquired in positive ion mode; **(B)** PCA score plot acquired in negative ion mode; **(C)** OPLS-DA score plot generated in positive ion mode; **(D)** Permutation test for the OPLS-DA model established in positive ion mode; **(E)** OPLS-DA score plot generated in negative ion mode; **(F)** Permutation test for the OPLS-DA model established in negative ion mode.

For data acquired in both the positive- and negative-ion modes, metabolites with VIP values > 1 and *p* values below 0.05 were defined as significantly altered metabolites. As shown in Figures 8A, B, the overall distribution patterns of metabolites and the marked differences between the Blank and PECD groups were clearly observed under both detection modes. Following the initial screening, annotation through literature comparison and online database retrieval identified a total of 1,597 significantly differential metabolites between the Blank and PECD groups, including 691 upregulated and 906 downregulated metabolites. As illustrated in Figure 8C, these 1,597 differential metabolites were further subjected to pathway analysis using Metabo Analyst 6.0. Pathway relevance to potential targets was evaluated based on the Impact value, and pathways with an Impact value >0.20 were considered to be closely associated with potential target pathways. Accordingly, the pathways most prominently influenced were phenylalanine metabolism, caffeine metabolism, and arginine biosynthesis, as summarized in Table 5. Table 6 summarized nine key metabolites associated with these three metabolic pathways that were influenced by PECD.

**Figure 8:**
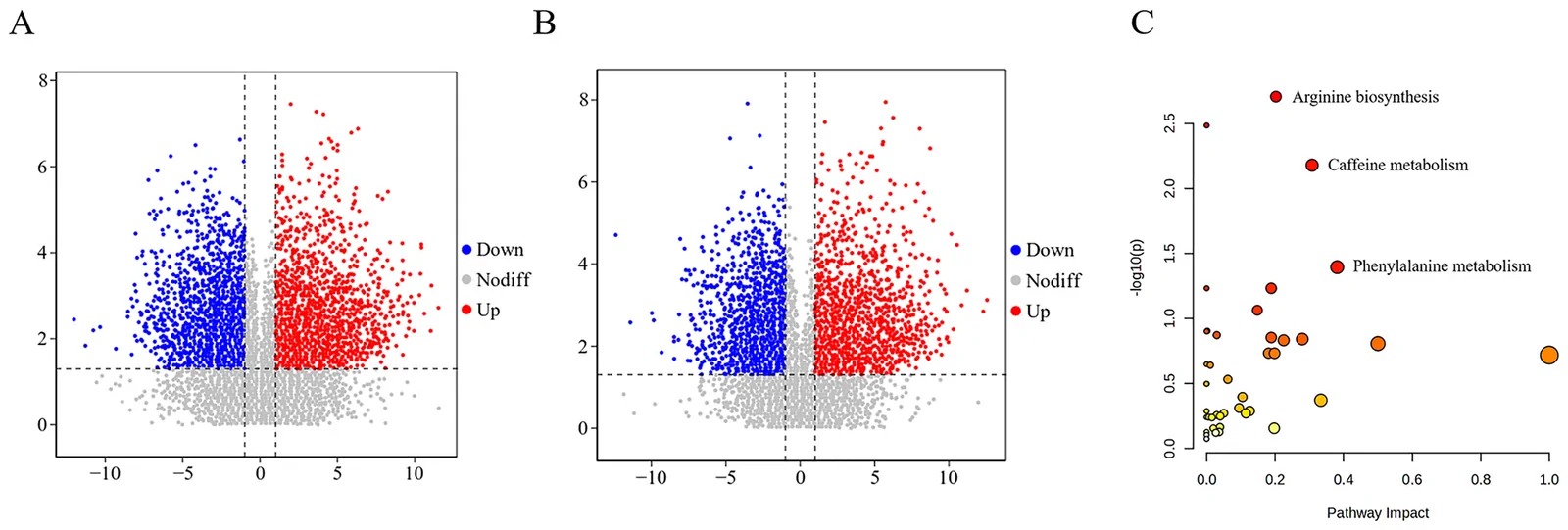
Volcano plot and Kyoto Encyclopedia of Genes and Genomes (KEGG) analysis of metabolites in Blank vs. PECD groups after 48 h of fermentation. **(A)** Volcano plot in positive mode. **(B)** Volcano plot in negative mode. **(C)** Pathway analysis of differential metabolites.

**Table 5:** Relevant information on three important metabolic pathways.

Pathway name	Match status	*p*	–log(*p*)	Holm *p*	False discovery rate (FDR)	Impact
Arginine biosynthesis	14/4	0.0019724	2.705	0.1578	0.13138	0.20213
Caffeine metabolism	10/3	0.0066295	2.1785	0.5171	0.17679	0.30769
Phenylalanine metabolism	8/2	0.040333	1.3943	1	0.7808	0.38096

**Table 6:** Nine important metabolites related to three significantly different metabolic pathways affected by PECD.

Name	Mode	*P* value	VIP	RT [min]	m/z	Regulation
1-Methylxanthine	–	0.034915384	1.424799761	1.586	165.04041	Up
N-Acetylornithine	–	0.003684313	1.527298963	0.681	173.09186	Up
7-Methyluric acid	–	0.001485071	1.526807607	1.259	181.03522	Down
Phenylacetaldehyde	+	0.007634162	1.526302864	4.606	121.06505	Up
Phenethylamine	+	0.022609904	1.464970433	2.901	122.09679	Down
L-Glutamate	+	3.73E−07	1.553580788	0.959	130.05038	Up
L-Arginine	+	0.001798623	1.53932648	0.639	175.1194	Up
7-Methylxanthine	+	0.022940074	1.477801265	1.579	167.05656	Up
N-Acetyl-L-glutamic acid	+	0.009538041	1.521491287	0.995	190.07146	Up

#### 3.3.7 Correlation analysis of gut microbiota and metabolites

Metabolite-dependent interactions between host systems and the intestinal microbiota are essential for maintaining physiological homeostasis. Microbiota-derived metabolites participate not only in local processes such as nutrient sensing, barrier maintenance, and immune regulation, but also in systemic metabolic signaling affecting distal organs (Kim et al., 2024). Previous research has confirmed that *Poria cocos* oligosaccharides could alleviate diet-induced glucose and lipid metabolic disorders in mice by regulating intestinal microbial composition (Zhu et al., 2022). Therefore, integrative correlation analysis between differential microbial taxa and altered metabolites is essential for functionally interpreting intervention-associated remodeling of the intestinal ecosystem (Wei et al., 2021).

To clarify the relationships between microbial taxa markedly affected by PECD fermentation and the corresponding metabolite alterations, Spearman correlation analysis was subsequently conducted. As shown in Figure 9, there is a clear correlation between different taxonomic groups and metabolites. Specifically, metabolites such as 1-methylxanthine, 7-methylxanthine, N-acetylornithine, L-glutamate, L-arginine, N-acetyl-L-glutamate, and phenylacetaldehyde showed significant positive correlations in gut microbiota such as *Escherichia, Bifidobacterium*, and *Mitsuokella*, and significant negative correlations in microbiota such as *Klebsiella, Bacteroides_H*, and *Bilophila*. However, 7-methyluric acid and phenethylamine showed opposite trends. These correlative patterns suggest that PECD-associated microbial shifts were linked to coordinated alterations in multiple metabolites converging on specific metabolic pathways, rather than isolated changes in individual metabolites. Notably, these metabolites were found to be primarily involved in metabolic pathways associated with phenylalanine metabolism, caffeine metabolism, and arginine biosynthesis. However, being derived from *in vitro* correlation analysis, these findings required functional validation.

**Figure 9:**
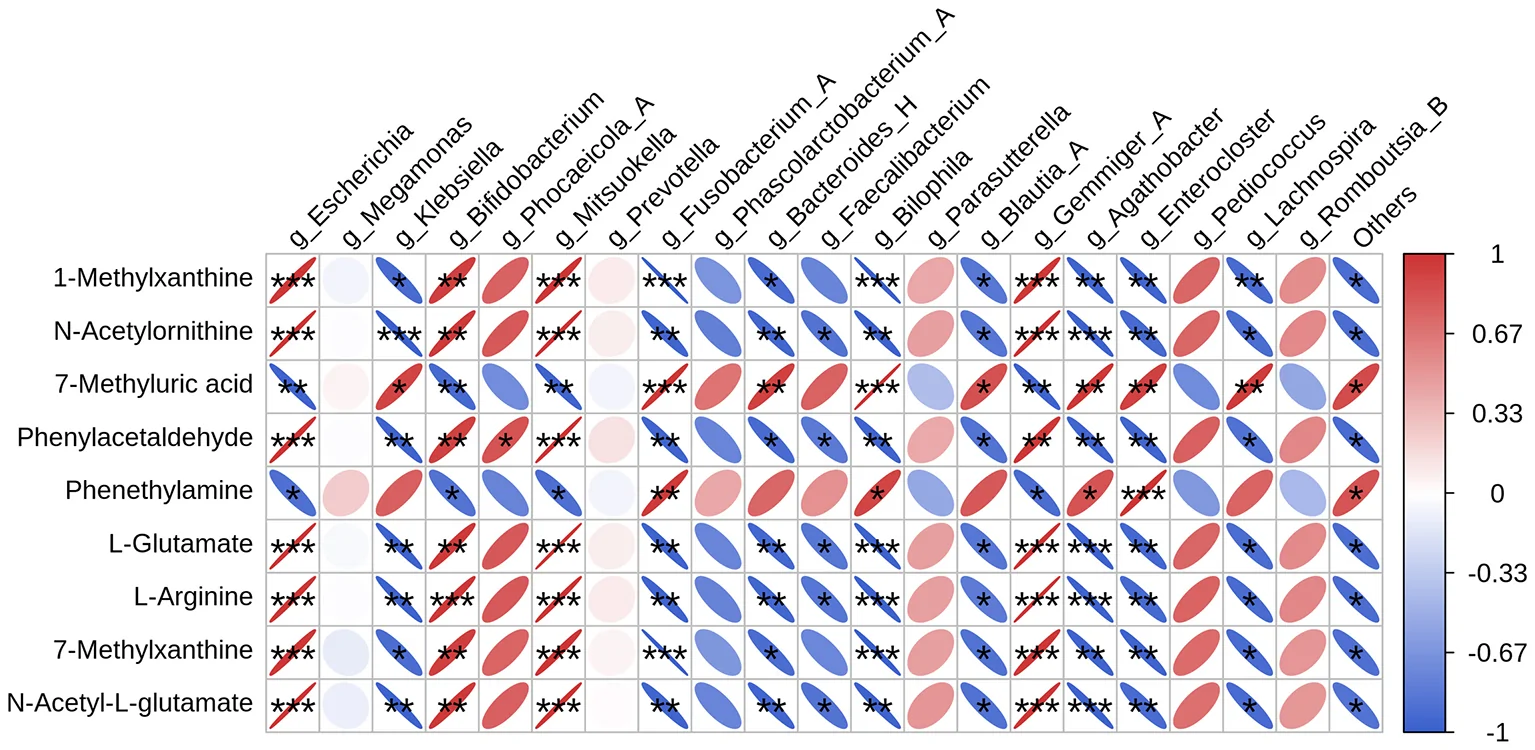
Correlation analysis between the altered gut microbiota and metabolites. *p < 0.05, **p < 0.01, ***p < 0.001.

## 4 Discussion

In this study, PECD remained highly stable throughout the simulated oral, gastric, and small-intestinal phases, with no significant changes observed in its monosaccharide composition, total carbohydrate content, reducing sugar content, or molecular-weight distribution. This indicated that the major polysaccharide components of PECD were resistant to hydrolysis by salivary enzymes, gastric acid, and intestinal digestive fluids under the conditions tested. For non-starch polysaccharides, resistance to digestion in the upper gastrointestinal tract is generally considered an essential prerequisite for reaching the colon and subsequently becoming available for utilization by the gut microbiota (Guo et al., 2021; Cao et al., 2024; Hu et al., 2024). The digestive resistance of PECD may be associated with its complex monosaccharide composition, structural heterogeneity and potential branching, glycosidic linkages that are not recognized by host digestive enzymes, and relatively restricted enzymatic accessibility. Monosaccharide analysis showed that PECD was composed of Man, GlcA, GalA, Glc, Xyl, Gal, Ara, and Fuc. In addition, the carboxyl-related characteristic absorption bands at approximately 1,741, 1,610, and 1,416 cm^−1^ in the FT-IR spectrum suggested the presence of uronic acid-containing or other acidic sugar domains. Furthermore, PECD could be partially degraded by β-galactosidase, glucanase, and pectinase in the PACE analysis, suggesting that it may contain GalA-enriched, pectin-like domains accompanied by neutral-sugar side chains (e.g., Ara, Gal, and Xyl) or other structurally distinct regions. Recent studies of non-starch polysaccharides derived from various medicinal and edible plants have similarly demonstrated that these polysaccharides may remain relatively stable during simulated gastrointestinal digestion but can subsequently be utilized by gut microorganisms upon entering colonic fermentation systems (Guo et al., 2021; Cao et al., 2024; Hu et al., 2024).

The subsequent *in vitro* fermentation behavior of PECD further suggested that its structural heterogeneity may simultaneously affect microbial utilization patterns and the kinetics of SCFAs production. Microbial degradation of dietary polysaccharides is jointly regulated by multiple factors, including molecular weight, monosaccharide composition, glycosidic-linkage pattern, backbone and side-chain organization, degree of branching, physicochemical accessibility, and the carbohydrate-active enzyme repertoire of the microbial community (Cao et al., 2024; Lee et al., 2024). Dietary polysaccharides with different microstructures may also induce distinct microbiota compositions and colonic mucin O-glycosylation responses, indicating that polysaccharide structure influences not only fermentability but may also contribute to broader host-microbiota interactions (Zhao et al., 2023). PACE fingerprint of PECD differed markedly from that of FOS, suggesting that PECD contained more heterogeneous carbohydrate fragments and structural domains. Utilization of complex pectin-like polysaccharides generally requires the coordinated action of multiple enzyme classes, including glycoside hydrolases, polysaccharide lyases, and carbohydrate esterases. It may also involve metabolic cooperation between primary degraders capable of cleaving intact polysaccharides and secondary consumers that utilize the released oligosaccharides, monosaccharides, or fermentation intermediates (Lee et al., 2024; Solvang et al., 2025). Therefore, the sustained utilization of PECD in the fermentation system may be related to the differential accessibility of individual structural domains, progressive depolymerization, and cross-feeding of the released products among different microbial populations.

PECD significantly decreased the pH of the fermentation system and supported the continuous accumulation of SCFAs, indicating that it promoted active microbial carbohydrate metabolism. Notably, pH is not merely an endpoint indicator of fermentation but also an important ecological factor influencing microbial community composition and metabolic output. Walker et al. found that adjusting the pH of continuous human fecal microbial cultures from 6.5 to 5.5 markedly altered both community composition and SCFA-production patterns (Walker et al., 2005). Therefore, the progressive acidification observed during PECD fermentation may have resulted primarily from carbohydrate utilization and organic-acid accumulation, while the increasingly acidic environment may, in turn, have affected microbial competition and metabolite exchange. Studies of other edible fungal and medicinal-food polysaccharides have similarly reported limited degradation during upper-gastrointestinal digestion, followed by polysaccharide depolymerization, decreasing pH, and increased SCFA production during fermentation (Hu et al., 2024; Jiang C. et al., 2024). Compared with FOS, PECD exhibited slower but more sustained fermentation and SCFA-production kinetics. This difference may be related to the more complex monosaccharide composition, heterogeneous glycosidic linkages, potential branching, and domain-dependent microbial accessibility of PECD. FOS, by contrast, has a relatively low degree of polymerization and a comparatively uniform fructan structure and can therefore generally be rapidly utilized by microorganisms possessing the corresponding fructan-utilization systems. PECD degradation may require a broader repertoire of carbohydrate-active enzymes and cooperative interactions among multiple microbial taxa. Spatially accessible terminal sugar residues, peripheral side chains, or exposed local domains may be preferentially utilized during the early stage of fermentation, whereas less accessible structural regions or linkages for which the corresponding enzyme systems are scarce may be degraded more slowly.

The dynamic changes in reducing sugar content further indicated that PECD degradation and microbial consumption did not proceed as a simple linear process. During the early stage of fermentation, cleavage of polysaccharide chains may generate new reducing ends and soluble carbohydrate fragments. As these products are subsequently taken up and utilized by microorganisms, their concentrations may decrease. When these processes occur concurrently, the reducing sugar content may show transient increases, fluctuations, or delayed decreases. Meanwhile, the gradual reduction in high-molecular-weight components, together with the persistence of the principal molecular-weight peak and major FT-IR absorption features after 48 h of fermentation, indicated that PECD mainly underwent localized and incomplete depolymerization rather than rapid, homogeneous, and exhaustive degradation. The released oligosaccharides and monosaccharides may also serve as substrates for other community members and thereby contribute to SCFA production during the later stages of fermentation. Taken together, progressive and domain-dependent depolymerization may represent an important structural basis for the sustained microbial utilization of PECD.

The structural differences between PECD and FOS, as evidenced by their distinct PACE fingerprints, provide a reasonable explanation for their divergent fermentation kinetics and microbial selectivity. The complex glycosidic architecture of PECD likely renders it less accessible to rapid microbial attack, resulting in slower but more sustained SCFA production and favoring the enrichment of *Bifidobacterium* and *Mitsuokella*. In contrast, the simpler structure of FOS allows rapid fermentation, preferentially promoting *Megamonas* and *Selenomonadaceae*. These differential features thus support the notion that glycosidic complexity governs substrate preference and ecological niche partitioning among gut microbes. At the taxonomic level, PECD fermentation was associated with increased relative abundances of *Actinomycetota, Bifidobacteriaceae*/*Bifidobacterium*, and *Mitsuokella*, together with decreased relative abundances of *Klebsiella* and *Bilophila*, indicating that different microbial taxa responded differently to PECD fermentation. The decrease in the relative abundance of *Bilophila* is noteworthy because previous studies have shown that *Bilophila wadsworthia* can aggravate inflammation, intestinal barrier dysfunction, bile-acid dysmetabolism, impaired glucose metabolism, and hepatic steatosis in high-fat-diet mouse models (Natividad et al., 2018). However, a genus-level decrease in relative abundance observed in an *in vitro* system is insufficient to demonstrate that PECD exerts a protective physiological effect. Similarly, the biological significance of the changes in these taxa requires further clarification using absolute quantification and functional analyses at the species or strain level. Following PECD fermentation, the relative abundance of *Escherichia* increased, and LEfSe analysis further identified *Pseudomonadota, Enterobacterales*_A, and *Gammaproteobacteria* as characteristic taxa. The genus *Escherichia* contains commensal, opportunistic, and pathogenic strains with markedly different functional properties. In the inflamed intestine, host-derived nitrate can confer a competitive growth advantage on *Escherichia coli* (Winter et al., 2013). However, the present *in vitro* fermentation system lacked the intestinal epithelium, immune-derived inflammatory mediators, and host-generated nitrate. Therefore, the increased relative abundance of *Escherichia* may reflect direct or indirect utilization of fermentation substrates, the persistence advantage of facultative anaerobes under the culture conditions, or compositional redistribution resulting from decreases in other microbial taxa. Future studies focusing on the complete structural elucidation of PECD, together with absolute quantification and functional validation at the strain level, will further refine our understanding of these structure-activity relationships.

PECD fermentation appeared to be primarily associated with the modulation of three metabolic pathways, including phenylalanine metabolism, caffeine metabolism, and arginine biosynthesis. Phenyalanine metabolism mainly involves the conversion of aromatic amino acids into aromatic aldehydes and biogenic amines. At the same time, it was found that L-phenylalanine in the phenylalanine metabolism pathway has been recognized as an important metabolic checkpoint for Th2 cell differentiation (Kulkarni et al., 2025), and metabolomics studies on acute exacerbation of childhood asthma have also shown that phenylalanine metabolism is one of the significantly abnormal pathways (Cottrill et al., 2023). Therefore, this metabolic pathway may be related to immune homeostasis regulation. Caffeine metabolism is another prominent pathway. The opposite correlation patterns of 1-Methylxanthine, 7-Methylxanthine, and 7-Methyluric acid suggest that PECD intervention may alter the metabolic direction of methylxanthine between demethylation, oxidation, and terminal transformation. Previous evidence suggests that elevated levels of caffeine metabolites are correlated with a reduced risk of current asthma and improved pulmonary function (Han et al., 2022), implying that this metabolic pathway may be involved in the regulation of airway inflammatory status. In addition, Arginine biosynthesis involves N-acetyllornithine, L-Glutamate, L-Arginine, and N-acetyl-L-glutamate, and their consistent correlation patterns indicate that Arginine biosynthesis is a metabolic focus (Cottrill et al., 2023). Due to the close relationship between arginine metabolism and nitric oxide production, redox balance, and mucosal inflammation, as well as significant abnormalities in arginine metabolism/arginine biosynthesis reported in asthma related metabolomics studies (Chen et al., 2025; Crestani et al., 2025), our correlative findings suggest that PECD-associated shifts in microbiota and nitrogen-containing metabolites may influence inflammatory homeostasis. However, these results were preliminary and required functional validation in disease models before any therapeutic implications could be drawn.

Nevertheless, several limitations of this study should be acknowledged, along with corresponding future directions. First, FITC tracing only reflects overall gastrointestinal localization and cannot exclude partial degradation or absorption of low-molecular-weight fragments; radiolabeling or pharmacokinetic analyses could provide more definitive tracking in future studies. Second, all experiments were conducted using healthy donors and mice, so the findings may not be generalizable to disease states such as phlegm-dampness syndrome, asthma, or obesity; validation in disease models and patient fecal samples is needed. Third, the use of pooled fecal inocula masks inter-individual variability, and batch fermentation lacks the dynamic gut environment (e.g., peristalsis, mucus, host signals); future studies using individual donor feces and continuous fermentation or gnotobiotic models are warranted. Fourth, 16S rRNA sequencing provides relative abundance rather than functional data, and our multi-omics correlations do not establish causality; functional validation via metagenomics, metatranscriptomics, fecal microbiota transplantation (FMT), or gene knockout studies is required to confirm causal relationships. Fifth, the animal distribution experiment used only three mice per time point due to ethical considerations; confirmatory studies with larger sample sizes are needed. Finally, our *in vitro* findings, including increased SCFAs, suggest prebiotic-like potential, but whether they translate into *in vivo* benefits (e.g., immune regulation, metabolic improvement, gut barrier enhancement) remains unknown. Thus, future studies integrating disease-relevant animal models, parallel controls of individual herb polysaccharides, and host functional endpoints are required to clarify the physiological relevance and structure-microbiota-metabolite relationships of PECD.

## 5 Conclusions

In conclusion, PECD exhibited substantial resistance to simulated upper-gastrointestinal digestion and underwent progressive and incomplete depolymerization during *in vitro* fecal fermentation. Compared with FOS, PECD showed slower and more sustained fermentation, prolonged SCFA production, and a distinct pattern of microbial community restructuring. These characteristics may be related to its heterogeneous structure and its requirement for multiple microbial utilization pathways. PECD fermentation was associated with changes in the relative abundances of genera including *Bifidobacterium, Mitsuokella, Klebsiella, Bilophila*, and *Escherichia*, as well as alterations in the overall microbial community structure and metabolite profile. Nevertheless, these findings primarily reflect compositional and metabolic responses within a closed *in vitro* system. Based on the currently available evidence, PECD is more appropriately described as a polysaccharide-enriched fraction with digestive resistance, fermentability, and microbiota-modulating potential. Taken together, these preliminary observations tentatively suggest that PECD may exert microbiota-mediated effects, and they encourage further exploration of its potential as a candidate bioactive polysaccharide, though additional *in vivo* studies are warranted to substantiate these findings.

## Data Availability

The raw data of this study are derived from the Mendeley data portal (https://data.mendeley.com/), which are publicly available under doi: 10.17632/fpbfjtk52p.1.
