# Supplementary material for: Polysaccharides from Er Chen Decoction: gastrointestinal fate, gut microbiota modulation and metabolomics
Source: Front Microbiol. 2026 Sep 15;17:1931914. doi: 10.3389/fmicb.2026.1931914 (PMC13619924; doi:10.3389/fmicb.2026.1931914)
Supplement: Supplementary file 1 [file Supplementary_file_1.docx]

**1. Structural characterization of PECD**

Monosaccharide composition analysis identified Man, Rha, GlcA, GalA, Glu, Gal, Ara, and Fuc as the predominant constituents of PECD, with molar ratios of 0.190 : 0.037 : 0.017 : 0.231 : 0.538 : 0.301 : 0.398 : 0.015, respectively (Fig. S1A). HPGPC analysis revealed that PECD comprised three fractions (Fig. S1B), with Mw values of 10471.65, 575.22, and 3.84 kDa, and Mn values of 4751.85, 521.12, and 1.28 kDa, respectively. The FT-IR profile of PECD exhibited several typical absorption signals (Fig. S1C). In particular, the broad absorption band centered at 3403 cm⁻¹ was attributed to O–H stretching, which suggests the existence of hydrogen-bonding interactions. An absorption peak near 2933 cm⁻¹ was assigned to C–H stretching in methylene moieties. In addition, the absorption band located at 1741 cm⁻¹ was attributed to the C=O stretching of esterified carboxyl groups, which may be related to glucuronic acid residues. The band around 1650 cm⁻¹ was considered characteristic of asymmetric C=O stretching vibrations. The band at 1427 cm⁻¹ was assigned to O–H bending vibrations, while the absorption peak at 1236 cm⁻¹ corresponded to C–O stretching vibrations. Furthermore, the region spanning 1200 to 1000 cm⁻¹, exhibiting peaks at 1137, 1090, and 1019 cm⁻¹, these absorption features were indicative of C–O–C and C–O–H vibrational modes within the pyranose ring structure. In addition, the characteristic signals at 855 and 762 cm⁻¹ suggested the presence of α- and β-glycosidic linkages, respectively. The UV spectrum of PECD (Fig. S1D) showed only weak absorbance in the 260–280 nm region, suggesting negligible protein/nucleotide contamination. This low background absorbance was consistent with the predominantly polysaccharide composition of the sample.

To further investigate the structural characteristics of PECD, we performed Polysaccharide Analysis using Carbohydrate gel Electrophoresis (PACE) following partial acid hydrolysis and specific glycosidase digestion. The results (Fig. S2) showed that untreated PECD exhibited no detectable bands, whereas FOS displayed distinct bands migrating between disaccharides and pentasaccharides. This indicated that PECD did not contain low-molecular-weight oligosaccharides and likely possesses a larger molecular size, a finding consistent with the molecular weight distribution results. Upon partial acid hydrolysis (Fig. S2A), both PECD and FOS generated oligosaccharide fragments. However, their banding patterns differed markedly from each other, suggesting that the acid‑labile glycosidic linkages in PECD and FOS were distributed differently. In contrast, after enzymatic digestion with β‑galactosidase, glucanase, or pectinase (Fig. S2B - D), PECD produced clear fragment bands, while FOS showed no detectable changes compared with its untreated form, indicating that PECD contains specific glycosidic bonds (e.g., β‑galactosyl, glucosyl, and galacturonosyl bonds) susceptible to these enzymes, whereas FOS is resistant to them. Collectively, these results demonstrated that PECD and FOS possessed fundamentally different structural architectures, which may serve as the structural basis for their distinct functional properties.

**Fig. S1.** Structural characterization of PECD. (A) HPGPC chromatogram of PECD. (B) HPLC chromatogram of PECD. The peaks of 1-9 were standards of Man, Rha, GlcA, GalA, Glc, Xyl, Gal, Ara, and Fuc, respectively. (C) FI-IR spectrum of PECD. (D) UV Spectral Scanning of PECD.

**Fig. S2.** Polysaccharide Analysis using Carbohydrate gel Electrophoresis of PECD. (A) – (D) were PACE images of PECD after partial acid hydrolysis by trifluoroacetic acid, β-galactosidase digestion, glucanase digestion, and pectinase digestion, respectively. 1. negative control (acid or enzymes only); 2. PECD after partial acid hydrolysis or enzymatic digestion; 3. FOS after partial acid hydrolysis or enzymatic digestion; 4. oligosaccharide standards (from bottom to top were glucose, maltose, maltotriose, maltotetraose, maltopentaose, maltohexaose, and maltoheptaose, respectively); 5. PECD without partial acid hydrolysis or enzymatic digestion; 6. FOS without partial acid hydrolysis or enzymatic digestion.

**2. Molecular weight parameters of PECD during fecal fermentation**

To quantitatively assess the molecular weight changes of PECD during *in vitro* t fecal fermentation, HPGPC analysis was performed at different time points. The weight‑average molecular weight (Mw), number‑average molecular weight (Mn), and polydispersity index (PDI) were calculated based on the elution profiles. The results are summarized in Table S1.

**Table S1.** Molecular weight parameters of PECD during *in vitro* simulated gastrointestinal digestion and fecal fermentation

| **Fermentation time** | **Peak-1** | | | **Peak-2** | | |
| --- | --- | --- | --- | --- | --- | --- |
|  | **Mw/kDa** | **Mn/kDa** | **PDI** | **Mw/kDa** | **Mn/kDa** | **PDI** |
| 0 h | 9580.92 ± 9.07^a^ | 3360.53 ± 1.75^a^ | 2.851 ± 0.004^e^ | 10.63 ± 0.02^a^ | 2.60 ± 0.01^d^ | 4.090 ± 0.016^a^ |
| 6 h | 3859.92 ± 0.36^b^ | 856.95 ± 0.11^b^ | 4.504 ± 0.000^a^ | 4.50 ± 0.01^e^ | 2.03 ± 0.00^e^ | 2.212 ± 0.002^d^ |
| 12 h | 2330.81 ± 1.09^c^ | 597.47 ± 0.09^c^ | 3.901 ± 0.002^c^ | 6.10 ± 0.01^d^ | 2.63 ± 0.01^c^ | 2.315 ± 0.005^c^ |
| 24 h | 1603.49 ± 6.15^d^ | 365.28 ± 0.11^d^ | 4.390 ± 0.016^b^ | 7.47 ± 0.03^b^ | 2.88 ± 0.01^a^ | 2.597 ± 0.018^b^ |
| 48 h | 900.40 ± 0.56^e^ | 281.10 ± 0.04^e^ | 3.203 ± 0.002^d^ | 7.34 ± 0.01^c^ | 2.80 ± 0.01^b^ | 2.616 ± 0.003^b^ |

Values assigned the same letter are not significantly different (*p* > 0.05), whereas values assigned different letters differ significantly (*p* < 0.05).
